# c-di-GMP Regulates Various Phenotypes and Insecticidal Activity of Gram-Positive *Bacillus thuringiensis*

**DOI:** 10.3389/fmicb.2018.00045

**Published:** 2018-02-13

**Authors:** Yang Fu, Zhaoqing Yu, Shu Liu, Bo Chen, Li Zhu, Zhou Li, Shan-Ho Chou, Jin He

**Affiliations:** ^1^State Key Laboratory of Agricultural Microbiology, College of Life Science and Technology, Huazhong Agricultural University, Wuhan, China; ^2^NCHU Agricultural Biotechnology Center, Institute of Biochemistry, National Chung Hsing University, Taichung, Taiwan

**Keywords:** c-di-GMP, *Bacillus thuringiensis*, diguanylate cyclase, phosphodiesterase, motility, biofilm formation, virulence, transcriptome

## Abstract

C-di-GMP has been well investigated to play significant roles in the physiology of many Gram-negative bacteria. However, its effect on Gram-positive bacteria is less known. In order to more understand the c-di-GMP functions in Gram-positive bacteria, we have carried out a detailed study on the c-di-GMP-metabolizing enzymes and their physiological functions in *Bacillus thuringiensis*, a Gram-positive entomopathogenic bacterium that has been applied as an insecticide successfully. We performed a systematic study on the ten putative c-di-GMP-synthesizing enzyme diguanylate cyclases (DGCs) and c-di-GMP-degrading enzyme phosphodiesterases (PDEs) in *B. thuringiensis* BMB171, and artificially elevated the intracellular c-di-GMP level in BMB171 by deleting one or more *pde* genes. We found increasing level of intracellular c-di-GMP exhibits similar activities as those in Gram-negative bacteria, including altered activities in cell motility, biofilm formation, and cell-cell aggregation. Unexpectedly, we additionally found a novel function exhibited by the increasing level of c-di-GMP to promote the insecticidal activity of this bacterium against *Helicoverpa armigera*. Through whole-genome transcriptome profile analyses, we found that 4.3% of the *B. thuringiensis* genes were differentially transcribed when c-di-GMP level was increased, and 77.3% of such gene products are involved in some regulatory pathways not reported in other bacteria to date. In summary, our study represents the first comprehensive report on the c-di-GMP-metabolizing enzymes, their effects on phenotypes, and the transcriptome mediated by c-di-GMP in an important Gram-positive bacterium.

## Introduction

**Figure 1:**
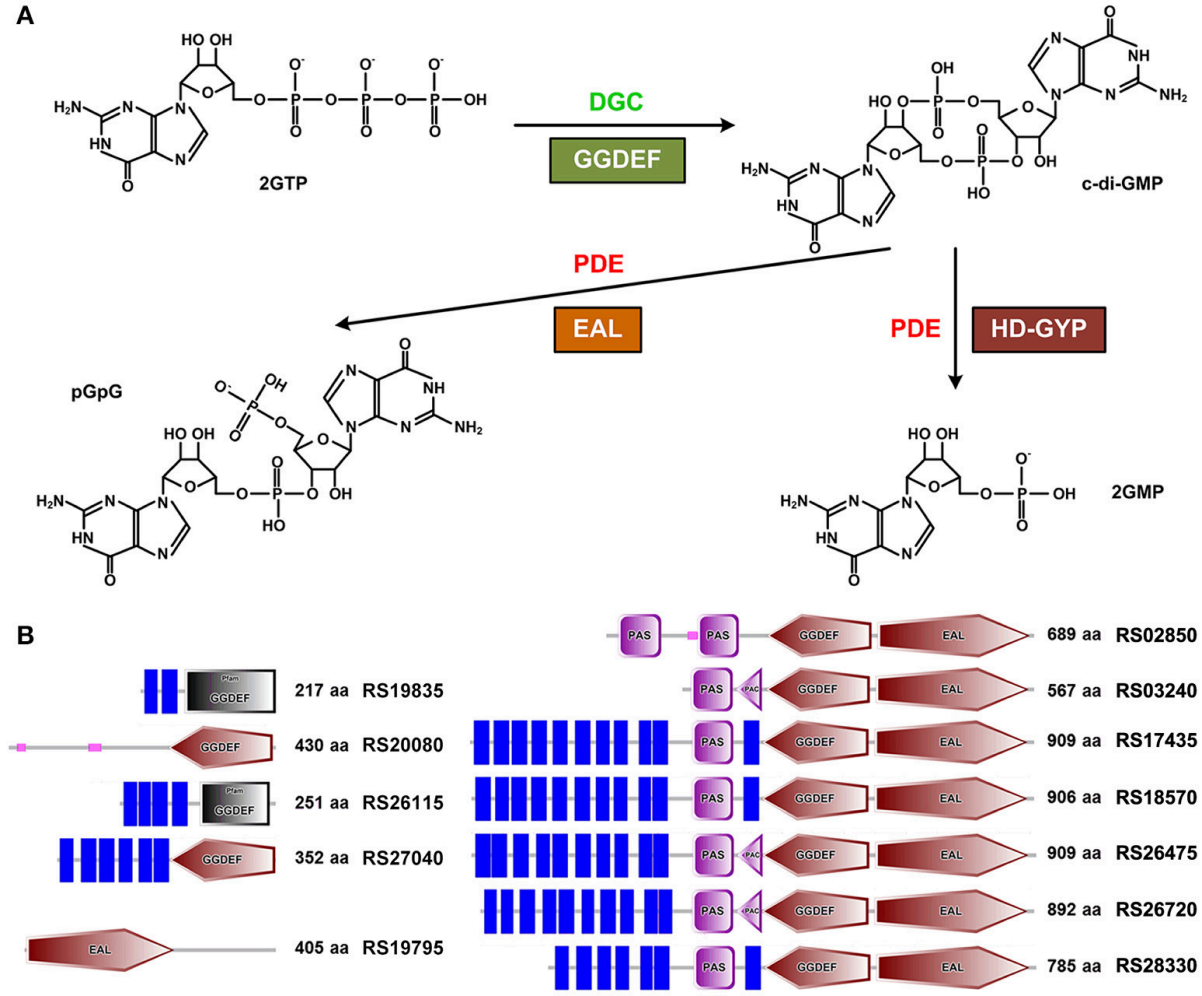
Identification of GGDEF, EAL, and HD-GYP domain-containing proteins in *B. thuringiensis* BMB171. **(A)** DGCs with the conserved GGDEF domain synthesize c-di-GMP using two molecules of GTP as substrate, while PDEs with EAL or HD-GYP domain degrade c-di-GMP to pGpG or two molecules of GMP (Rajeev et al., 2014), respectively. **(B)** Domain composition of the 12 putative proteins involved in c-di-GMP metabolism in BMB171. Four proteins contain an isolated GGDEF domain, one with an isolated EAL domain, and seven with a dual GGDEF-EAL domain. Predicted transmembrane regions are shown with blue rectangles. PAS domain (IPR000014) is a signal sensor domain (Taylor and Zhulin, 1999), while PAC domain (IPR001610) is proposed to help PAS domain folding (Zhulin et al., 1997). Domain symbols are taken from the SMART database. The ORF and number of amino acids in each protein are shown in the right side. Proteins RS19835 and RS26115 (black rectangles) are listed to contain a GGDEF domain in the Pfam database 28.0, but without the canonical GGDEF signature domain in the SMART database.

Bis-(3′-5′)-cyclic dimeric guanosine monophosphate (c-di-GMP) is a second messenger playing important roles in a plethora of bacterial physiological processes to suppress motility (Albert-Weissenberger et al., 2010; Boehm et al., 2010; Krasteva et al., 2010), mediate transition from planktonic growth to biofilm formation (Newell et al., 2009, 2011; Ha and O'Toole, 2015; Chen et al., 2016), coordinate with quorum sensing (Kariisa et al., 2016; Matsuyama et al., 2016) and alter virulence gene expression (Aragón et al., 2015; Kariisa et al., 2015; Suppiger et al., 2016) in many Gram-negative species, as well as to confer resistance to antibiotics and induce host immune responses (Aldridge et al., 2003). In general, the intracellular c-di-GMP level is controlled by diguanylate cyclases (DGCs) and phosphodiesterases (PDEs) for its synthesis and degradation, respectively. c-di-GMP is synthesized from two molecules of GTP by DGCs that usually encode a GGDEF domain (Ausmees et al., 2001; Chan et al., 2004). Degradation of c-di-GMP into one molecule of pGpG or two molecules of GMP is carried out by two distinct c-di-GMP-specific phosphodiesterases that contain a conserved EAL domain or a HD-GYP domain, respectively (Christen et al., 2005; Schmidt et al., 2005; Ryan et al., 2006; Rajeev et al., 2014; Figure 1A). The number of genes encoding for enzymes related to c-di-GMP turnover differs dramatically, ranging from one to dozens among different species through whole genome analysis for a diverse array of bacterial species (Kalia et al., 2013; Römling et al., 2013). For instance, *Mycobacterium smegmatis* was predicted to possess one protein with a dual GGDEF-EAL domain that exhibits both DGC and PDE activities (Bharati et al., 2012). In contrast, more than 60 potential genes associated with the c-di-GMP turnover were found in *Vibrio cholera* (Beyhan et al., 2008). In general, these c-di-GMP-metabolizing enzymes are believed to locate at different cellular positions, responding to different environmental stimulus at different growth phases to precisely regulate the intracellular c-di-GMP level in a temporal and spatial way. In addition, some eukaryotic strains (e.g., eukaryote *Dictyostelium discoideum* Chen and Schaap, 2016) also secrete c-di-GMP to keep c-di-GMP homeostasis.

c-di-GMP is believed to perform diverse regulatory functions by binding to a wide variety of receptors in bacteria (Chou and Galperin, 2016), including kinases or phosphorylases (Tuckerman et al., 2011; Lori et al., 2015), transcription factors (Chin et al., 2010; Tao et al., 2010; Sondermann et al., 2012), PilZ domain proteins (Amikam and Galperin, 2006; Yang et al., 2015; Zhu et al., 2017), MshEN- domain proteins (Wang et al., 2016a) and degenerate DGCs or PDEs (Lee et al., 2007; Abel et al., 2011; Sondermann et al., 2012; Römling et al., 2013). The other major class of c-di-GMP effectors is RNA riboswitch (Sudarsan et al., 2008; Shanahan et al., 2011; Bordeleau et al., 2015; Tang et al., 2016; Zhou et al., 2016; Li et al., 2017a), which is usually located in the 5'-UTR of mRNA to control gene expression in response to changing c-di-GMP concentrations.

As one of the most popular insecticides, *Bacillus thuringiensis* possesses insecticidal activity *via* production of insecticidal crystal proteins (ICPs) during sporulation (Bravo et al., 2011; He et al., 2011). Based on this unique feature, *B. thuringiensis* can be considered different to other *B. cereus* group species (Mei et al., 2015). Apart from ICPs, *B. thuringiensis* also harbors genes encoding other virulence factors including cytolytic parasporal toxins, enterotoxins, hemolysins, phospholipases and proteases similar to other *B. cereus* group species (Raymond et al., 2010; Raymond and Bonsall, 2013; Soberón et al., 2013; Palma et al., 2014; Xu et al., 2014). These virulence factors secreted by *B. thuringiensis* are toxic to certain host larvae such as *Lepidopterous* (Slamti et al., 2014). They possibly work by binding to certain specific receptors protein anchoring on the midgut epithelial cells to induce cell lysis, which can form a favorable condition for *B. thuringiensis* to multiply in the insect hemocoel (Palma et al., 2014), which result in septicemia (Lucena et al., 2014; Slamti et al., 2014). Recent advances in the studies of *B. thuringiensis* are gradually moving to revealing regulation of various virulence factors expression at the transcription and translation levels in order to make better use of *B. thuringiensis* as insecticides (Wang et al., 2013a,b; Deng et al., 2014).

Yet, how virulence factor production is regulated by c-di-GMP in the Gram-positive bacteria is, however, unclear to date. It would thus be very interesting to investigate the physiological changes caused by different c-di-GMP levels in the *B. cereus* group species. In this study, we have chosen the Gram-positive entomopathogenic *B. thuringiensis* as the target to verify the activities of all putative proteins with GGDEF and/or EAL domains in a systematic way. In this regard, we also constructed a high intracellular c-di-GMP level mutant by deleting the three c-di-GMP-specific PDE genes. Using this mutant, we further explored the phenotype changes mediated by increasing c-di-GMP level and comprehensively investigated gene expression changes at transcription level.

## Materials and methods

### Bacterial strains, plasmids, culture condition, and primers

The bacterial strains used for our study were listed in Table S1. *Escherichia coli* strains were routinely cultured in Lysogenic Broth (LB) medium at 37°C and *B. thuringiensis* strains were cultured in LB medium at 28°C. The growth curves of *B. thuringiensis* strains were determined in LB medium. Antibiotics were added at concentrations as the followings: ampicillin, 100 μg/mL; kanamycin, 50 μg/mL; spectinomycin, 300 μg/mL for *B. thuringiensis* and spectinomycin at 100 μg/mL for *E. coli*.

All restriction enzymes were purchased from Takara Corporation and DNA polymerase purchased from CWbio Corporation. The plasmids used in this study were listed in Table S1. The primers used for the vector and strain constructions for the experiment target were listed in Table S2. Genomic DNA from *B. thuringiensis* strain BMB171 (GenBank accession number NC_014171) was used as the template in all PCR experiments.

### Cloning and purification of putative proteins with GGDEF and/or EAL domains

The 12 genes encoding full-length or truncated proteins were amplified from BMB171 by PCR using corresponding primers for constructing expression vectors. All PCR products were digested using restriction enzymes 5′ *Nco*I and 3′ *Xho*I except for *RS26720*, the PCR product of which was cut using 5′ *Nco*I and 3′ *Hind* III and was inserted into the Novagen vector pET-28b(+) with the same restriction sites. The recombinant vector containing the correct sequence was then transformed into the *E. coli* BL21(DE3) competent cells for the overexpression of these 12 putative DGC and PDE proteins fused with a C-terminal His_6_-tag. The bacterial strains and plasmids used were listed in Table S1. The recombinant proteins with a C-terminal His_6_-tag were purified using the Ni-NTA resin as previously reported (Yang et al., 2010). The purity of the target protein was checked using a 12% sodium dodecylsulfate-polyacrylamide gel electrophoresis (SDS-PAGE), with the concentration measured by the Bradford method using bovine serum albumin as the standard (Zheng et al., 2013).

### *In vitro* enzymatic activity assays

To detect DGC activity, the proteins with a GGDEF domain (10 μM) were mixed with 100 μM GTP in 50 mM Tris-HCl (pH 7.6), 50 mM NaCl, and 20 mM MgCl_2_ to a total volume of 100 μL. For PDE activity detection, each protein with an EAL domain (10 μM) was mixed with 50 μM c-di-GMP in 50 mM Tris-HCl (pH 7.6), 50 mM NaCl, 20 mM MgCl_2_, and 5 mM MnCl_2_ to a total volume of 100 μL. The reaction mixture was incubated at 4°C for 12 h, and then stopped by heating in a boiling water bath for 15 min. The mixture was further centrifuged at 14,000 × g for 15 min at 4°C, with the supernatant loaded onto a Waters 1525 HPLC Breeze system (wavelength was set as 254 nm). Consecutively, the supernatant was purified using an Elite Hypersil BDS C18 column (200 × 4.6 mm; 5 μm particle sizes) with a mobile phase of 20 mM ammonium acetate solution containing 10% methanol. The column temperature was kept at 25°C, and the flow rate was set at 1 mL/min.

### Q-TOF analysis

The reaction product was concentrated by vacuum freezing for 28 h, and was resuspended in 200 μL ddH_2_O. The sample was further centrifuged at 14,000 × g for 15 min and the supernatant was loaded onto an Agilent HC-C18 column (250 × 4.6 mm; 5 μm particle sizes) in the Agilent 6540 Ultra High Definition (UHD) Accurate-Mass Q-TOF LC-MS system (wavelength was set as 254 nm). The MS operating conditions were capillary Voltage 3500 V, nebulizer pressure 35 psig, the flow rate of drying gas 9 L/min and temperature 350°C. The scanning range of Q-TOF was *m/z* 100–1,000 under the negative-ion detection mode. Data were acquired at the rate of one spectrum per second (Xin et al., 2015).

### Construction of mutants using markerless gene deletion method

The upstream and downstream sequences of the target genes were amplified from BMB171 by PCR using the primer pairs (Table S2) for mutant construction. Previously published methods were used to screen the correct single colony as shown in Figure S1 and the correct clone was detected using PCR method as reported (Janes and Stibitz, 2006; Zheng et al., 2015).

### Quantification of intracellular c-di-GMP concentration by LC-MS/MS

BMB171 and Δ3*pde* were cultured at 28°C for 9 and 48 h, respectively. The cells were then harvested (100 mL cultures) by centrifugation at 4°C. The cell pellets were used for c-di-GMP extraction as previously reported (Spangler et al., 2010; Tang et al., 2015) with a few modifications. Detection of c-di-GMP was performed using a Finnigan Surveyor Plus liquid chromatography system followed by a Thermo Scientic TSQ Quantum Ultra EMR tandem mass spectrum system (San Jose, USA) using a selected reaction monitoring mode with the following m/z transitions: 691.135/135.100 at 51 eV, 691.135/152.000 at 39 eV and 691.135/248.300 at 25 eV. The daughter ion 152.000 was selected as the quantitative ion.

### Quantitative transcriptomics (RNA-seq)

BMB171 and Δ3*pde* were cultured at 28°C for 9 h. Total was isolated with the TRIzol LS Reagent (Ambion, USA). After proper processing, each sample was sequenced using a paired-end strategy by Illumina Hiseq™2500 (Illumina, USA). The transcriptome data were obtained by strand-specific RNA-seq using the Illumina Genome Analyzer IIx sequencing platform (Li et al., 2017b). The raw data of each sample was collected and filtered by the FASTX-Toolkit (http://hannonlab.cshl.edu/fastx_toolkit/) to get a clean data set that can be further analyzed and processed. The average length of the clean-reads was 100 nt, and the total numbers of clean-reads reached 3,876,126 and 3,937,473 for the libraries of Δ3*pde* and BMB171, respectively. The clean-reads of every sample were then mapped to unigene of reference genome using Bowtie2 (version 2.2.3) with a threshold e value of 0.00001 and the “-N <1>” parameter (Langmead and Salzberg, 2012), which allowed mapping of reads to the genome down to one mismatch. Reads mapped to rRNA were excluded from further analysis. The number of reads mapped to each gene was recorded by R (Mortazavi et al., 2008) and normalized into RPKM (Reads Per Kilo bases per Million reads). The differentially expressed genes were recorded by the DEGseq package using the MARS (MA-plot-based method with Random Sampling model) method (Wang et al., 2010). We used FDR ≤0.001 and a ≥1.5-fold change (|log1.5 (Fold change) normalized| ≥1) as the threshold to judge the significance of gene expression difference. These differentially expressed genes were assigned to functional category by the KOBAS (KEGG Orthology Based Annotation System) 3.0 Program (http://kobas.cbi.pku.edu.cn) and annotated from NCBI.

### RT-qPCR (reverse transcription quantitative PCR)

Primers were designed using the Primer 5.0 tool and tested for unique binding by Primer- BLAST (Ye et al., 2012; Table S2). Previous published methods for RNA extraction and calculation of relative transcription level for target genes were employed (Wang et al., 2015). The gene transcription levels were quantified relative to the transcription of reference gene *gapdh* (glyceraldehyde 3-phosphate dehydrogenase) (Løvdal and Saha, 2014) and the relative quantification of genes was visualized using Origin 8.0 software.

### Extraction and determination of Cry1Ac10 protein

Shuttle plasmid pBMB43-304 (Qi et al., 2015) encoding the ORF of *cry1Ac10* was transformed (electroporation) into BMB171 and Δ3*pde* to generate BMB171-*cry1Ac* and Δ3*pde*-*cry1Ac* strains, respectively which were grown in LB medium at 28°C and 200 rpm conditions. At the indicated time point (48 h) (Figure S2), 20 mL of each culture was separately harvested. The previously reported procedure for extracting Cry1Ac10 protein was followed (Wang et al., 2016b). The concentration of Cry1Ac10 protein was further determined using the Bradford method with the purity checked by SDS-PAGE.

### Cell motility assays

All examined strains were grown under the same initial inoculum concentration and culture conditions, shaken for 9 h at the mid-log phase, before cell motility assays. Swimming agar plates (LB semisolid medium with 0.5% agar) were dried for 10 min in a laminar flow hood before use. Five microliter liquid bacteria culture from the strains was then spotted to certain location of the plates, which were dried for another 10 min and incubated at 28°C for 8 h.

### Biofilm formation assays

The strains were shaken and grown in LB medium for 12 h before static growth for another 36 h. The ability of biofilm formation of the strains was determined by an improving method as reported for *Clostridium difficile* (Bordeleau et al., 2011). Biofilm formation assay was performed at least for three independent experiments.

### Cell-cell aggregation assay

A positively charged cell slide WHB-48-CS (Solarbio, China) was put into the cell culture tube. The special slide was treated by tissue culture with positive charge, enabling bacteria with negative charge to grow adherent to the slide. The strains were shaken and grown in LB medium for 6–8 h under the same culture conditions. The cell slides were taken out from culture tube and washed by phosphate buffer (pH 7.2) for three times, and then fixed with 2.5% (v/v) glutaraldehyde overnight at 4°C, followed by dehydration with ethanol at different gradient concentrations (30, 50, 70, 80, 90, and 100%). Consecutively, the cell slides were dried using critical point drying method. Cell morphologies were performed and imaged under a JSM-6390/LV scanning electron microscope (JEOL, Japan) with a 20,000 V accelerating voltage.

### Virulence assay

The strains were grown at selected time point under similar conditions. The cells were then harvested by centrifugation (10,000 × g, 5 min, 4°C) to obtain pellets that were resuspended in ddH_2_O. The strain to be assayed was added into an artificial diet (100 mL medium containing 4 g yeast extraction, 7 g bean meal, 0.5 g vitamin C, 1.5 g agar, 1.5 mL 36% acetic acid and 2 g penicillin) as additive (Tang et al., 2016). The artificial diet was transferred to 24-well trays (1 mL/well) (Costar, USA). 100 μL cultures of different *B. thuringiensis* strains were spotted on the diet. In every group, 72 larvae were transferred individually to 24-well culture plates. The survival rate was recorded daily for 7 days. The length and the weight of larvae were measured at the end of rearing cycle. Each data was collected for at least three replicates.

## Results

### Distribution of putative proteins with GGDEF and/or EAL domains in *B. thuringiensis* BMB171

Through searching the Pfam database (Finn et al., 2014) and NCBI Conserved Domains database (Marchler-Bauer et al., 2015) for proteins containing GGDEF, EAL and/or HD-GYP domains in the *B. cereus* group strains with complete genomes, we have accessed a list of proteins involved in the c-di-GMP metabolism as shown in Table S3. We noticed that there were approximately nine proteins containing the GGDEF and/or EAL domains and HD-GYP domain in each strain in the *B. cereus* group. In BMB171 (He et al., 2010), we accessed in total 12 putative proteins with the GGDEF and/or EAL domains using the SMART database (http://smart.embl-heidelberg.de/). These putative proteins include four with a GGDEF domain (RS19835, RS20080, RS26115, and RS27040), one with an EAL domain (RS19795) and seven with a dual domain (RS02850, RS03240, RS17435, RS18570, RS26475, and RS26720 in chromosome and RS28330 in the plasmid pBMB171) (Figure 1B). Among them, eight proteins are believed to be membrane-associated *via* SMART database, while the other four are distributed in the cytoplasm, demonstrating that c-di-GMP-metabolizing enzymes may act and function in different space inside *B. thuringiensis* BMB171 the cell.

### Determination of transcription levels and heterotrophic purification of the putative proteins with GGDEF and/or EAL domains

In order to investigate the transcription of genes coding for these putative proteins, we further determined their transcription levels at different growth phases *in vivo* as well as their enzyme activities *in vitro*, respectively. Firstly, we selected four different growth phases (at 9, 27, 48, and 72 h; Figure S2A) to carry out reverse transcription PCR to detect their cDNAs in BMB171. As shown in (Figure S3), five genes (*RS20080, RS19795, RS02850, RS03240*, and *RS18570*) were transcribed at relatively high levels at all four growth phases, four genes (*RS19835, RS27040, RS17435*, and *RS26720*) were only transcribed at some growth phases, while three genes (*RS26115, RS26475*, and *RS28330*) were in relatively low transcription levels at all four growth phases. Next, the 12 genes were heterogeneously expressed in *E. coli* BL21(DE3) with a His_6_-tag in their C-terminal domains and purified through a Ni-NTA affinity column. To obtain soluble proteins, three proteins (RS19835, RS26115 and RS27040) with a GGDEF domain (Figure S4A) and five proteins (RS17435, RS18570, RS26475, RS26720, and RS28330) with a dual GGDEF-EAL domain (Figure S4B) were purified with their transmembrane domains truncated. Except for RS26475 and RS28330, all the other proteins were obtained in soluble form, and the SDS-PAGE results demonstrated that all the proteins migrated at positions correlated with their theoretical MW (Figure S5).

### Determination of PDE activities of putative proteins with an EAL domain *in vitro*

**Figure 2:**
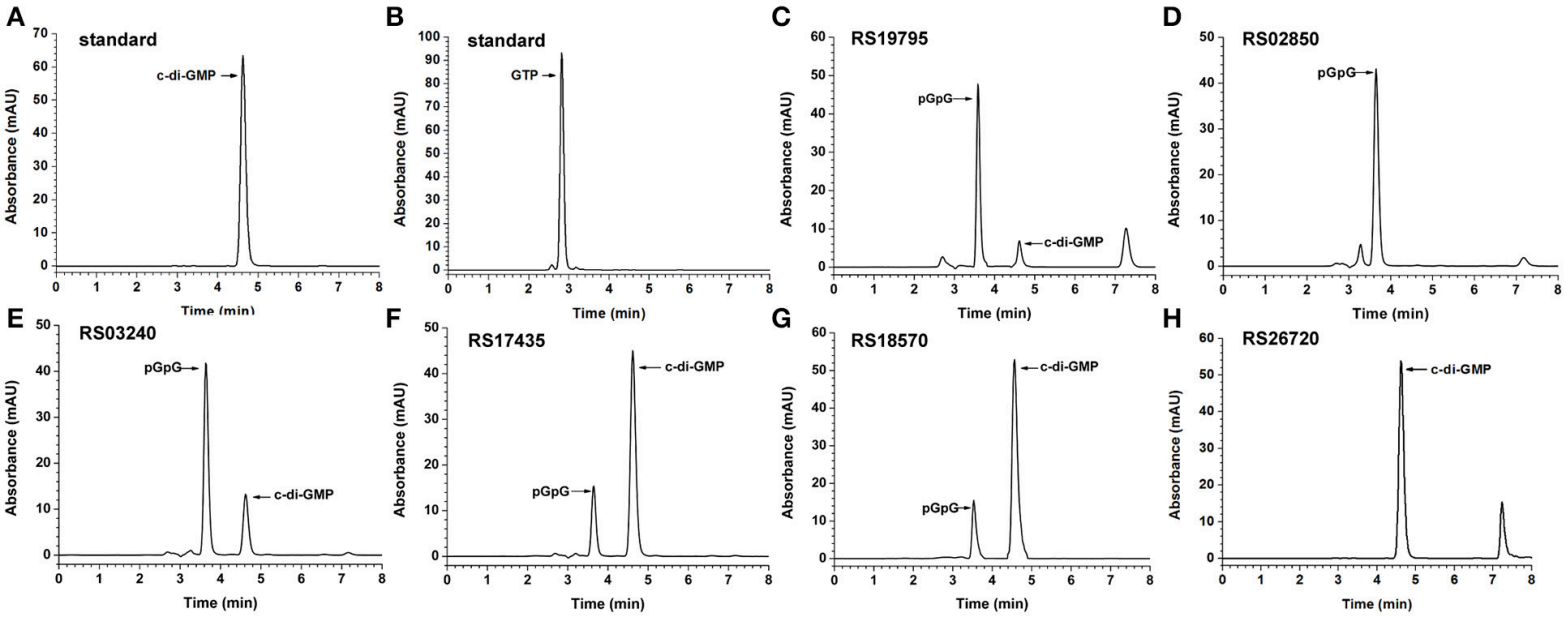
Determination of *in vitro* PDE activities of putative proteins with GGDEF and/or EAL domains. **(A–H)** The chromatograms of HPLC analyses shown for c-di-GMP standard, GTP standard, single EAL domain protein (RS19795), dual GGDEF-EAL domain proteins (RS02850 and RS03240), dual GGDEF-EAL domain proteins with the transmembrane region truncated (RS17435, RS18570, and RS26720), respectively.

The purified recombinant proteins containing an EAL domain were tested for their ability to degrade c-di-GMP *in vitro* by HPLC analysis (Figure 2). The HPLC chromatogram of RS19795 with an EAL domain showed a new product peak (Figure 2C) that was eluted before the peak of c-di-GMP (Figure 2A). Similarly, four other purified proteins (RS02850, RS03240, RS17435, and RS18570) with a dual GGDEF-EAL domain also showed a new product peak at the similar retention time as that of RS19795 (Figures 2D–G), but the dual domain protein RS26720 didn't show any new product peak (Figure 2H). In order to confirm the identity of the new peak, the reaction product of RS03240 was chosen for characterization by using a negative-ion mode LC-MS analysis (Xin et al., 2015; Figure S6). The results demonstrated that the new product peak at 3.57 min is consistent with the molecular ion peak of pGpG with an *m/z* of 707.2 [M-H]^−^ (Figure S6B) and the peak at 5.17 min is consistent with that of c-di-GMP with an *m/z* of 688.7 [M-H]^−^ (Figure S6C). These results indicated that the five proteins RS19795, RS02850, RS03240, RS18570, and RS17435 containing an isolated EAL domain or a dual GGDEF-EAL domain exhibit the expected PDE activity to hydrolyze c-di-GMP into pGpG.

In addition, we also used another cyclic-di-nucleotide signaling molecule c-di-AMP as the control substrate, and the result indicated that these putative proteins with a GGDEF domain and an EAL domain were inactive in degrading c-di-AMP.

### Determination of DGC activities of putative proteins with a GGDEF domain both *in vitro* and *in vivo*

**Figure 3:**
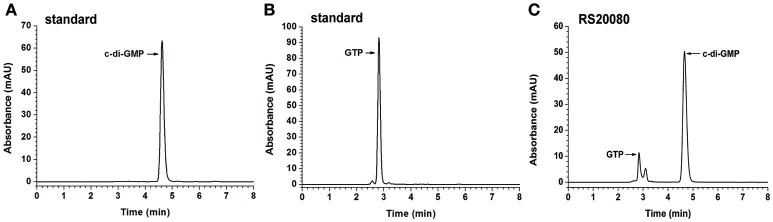
Determination of *in vitro* DGC activities of putative proteins with single GGDEF domain. The chromatograms of HPLC analyses shown for c-di-GMP standard **(A)**, GTP standard **(B)**, and single GGDEF domain protein RS20080 **(C)**, respectively.

The putative proteins with a single GGDEF domain (RS19835, RS20080, RS26115, and RS27040) were also tested for their ability to synthesize c-di-GMP *in vitro* (Figure 3 and Figure S7). The HPLC chromatogram of RS20080 (Figure 3C) showed a new product peak at the same retention time as that of the c-di-GMP standard (Figure 3A), indicating that RS20080 possesses the expected DGC activity with GTP molecule as substrate (Figures 2B, 3B). Meanwhile, protein RS27040 was also demonstrated to be a DGC yet with a lower activity (Zhou et al., 2016). Both RS19835 and RS26115, on the contrary, produced no c-di-GMP at all during the same reaction condition (Figures S7C,D). Similarly, proteins with a dual GGDEF-EAL domain (RS02850, RS03240, RS17435, RS18570, and RS26720) were also found to lack the capability of synthesizing c-di-GMP *in vitro* (Figures S7E–I).

To further verify their DGC activity *in vivo*, we designed a novel dual-fluorescence reporter system (Figure S8A; Zhou et al., 2016). Based on this system, RS27040 was verified to be an active DGC (Figures S8B,C). The dual-fluorescence reporter was further performed to determine the DGC activity of all other proteins with the typical GGDEF domain *in vivo*, including the six soluble proteins (RS20080, RS02850, RS03240, RS17435, RS18570, and RS26720) and the two insoluble proteins (RS26475 and RS28330) (Figure S8). These results indicated that protein RS20080 with a single GGDEF domain exhibited a high DGC activity and protein RS03240 showed a low DGC activity; but dual domain proteins RS02850, RS17435, RS18570, RS26720, RS26475, and RS28330 had no DGC activity at all (Figures S8B,C). In a word, RS20080 and RS27040 were demonstrated to possess DGC activity in both *in vitro* and *in vivo* assays; however, RS03240 could only be verified to exhibit a low DGC activity in the *in vivo* system, indicating that the *in vivo* system has advantage compared to the *in vitro* assays in revealing enzymatic activity.

Notably, RS03240 may be the only bi-functional protein exhibiting both DGC and PDE activities in BMB171. However, considering the fact that RS03240 exhibited a high PDE activity yet a low DGC activity, it may act preferentially as a PDE *in vivo* too.

### Intracellular c-di-GMP level was dramatically increased in Δ3*pde* compared to the parent strain BMB171

To explore the c-di-GMP signaling pathway in BMB171, we deleted genes encoding PDE to elevate the intracellular c-di-GMP concentration. In this regard, we selected four candidate genes (*RS02850, RS03240, RS18570*, and *RS19795*) with high transcription and high PDE activity *in vitro* to construct different mutants using the markerless gene deletion method (Janes and Stibitz, 2006; Zheng et al., 2015). According to the obvious phenotype changes (cell motility as the first step screening; data not shown), we cautiously selected the start gene and the subsequent gene to delete for rapid construction of high intracellular c-di-GMP level mutants. Through necessary screening, we obtained a positive mutant from the several constructed mutants including two single *pde* gene deletion mutants Δ*RS03240* and Δ*RS19795* (Figure S1B), two double *pde* genes deletion mutants Δ*RS03240*Δ*RS02850* and Δ*RS19795*Δ*RS02850* (Figure S1B), and one triple *pde* genes deletion mutant Δ*RS03240*Δ*RS02850*Δ*RS18570*. Interestingly, three mutants (Δ*RS03240*, Δ*RS03240*Δ*RS02850*, and Δ*RS03240*ΔRS02850ΔRS18570) exhibited a dosage-dependent phenotype phenomenon in cell motility, biofilm formation and aggregation rate. Unless indicated, the names of Δ1*pde*, Δ2*pde*, and Δ3*pde* will be used to refer to the Δ*RS03240*, Δ*RS03240*Δ*RS02850*, and Δ*RS03240*ΔRS02850ΔRS18570 mutants, respectively (Figure S1B).

To verify that these genes deletion did change the intracellular c-di-GMP level, we chose the Δ3*pde* and parent strain BMB171 for comparison to detect their intracellular c-di-GMP level at two different growth phases, i.e., the mid-exponential growth phase (9 h) and the sporulation phase (48 h) (Figure S2) by LC-MS/MS (Figure S9). The result showed that the intracellular c-di-GMP level of Δ3*pde* and BMB171 at 9 h were 339.0 fmol/mg and 29.9 fmol/mg, respectively. At 48 h, those of Δ3*pde* and BMB171 decreased to 16.7 and 4.8 fmol/mg, respectively. The c-di-GMP levels of Δ3*pde* mutant thus increased by 11.3- and 3.4-fold compared to parent strain BMB171 at the 9 h and 48 h growth phases, respectively, proving that the intracellular c-di-GMP level in Δ3*pde* is substantially elevated compared to the parent strain BMB171 at different growth phases.

### High intracellular c-di-GMP level inhibits cell motility of *B. thuringiensis*

**Figure 4:**
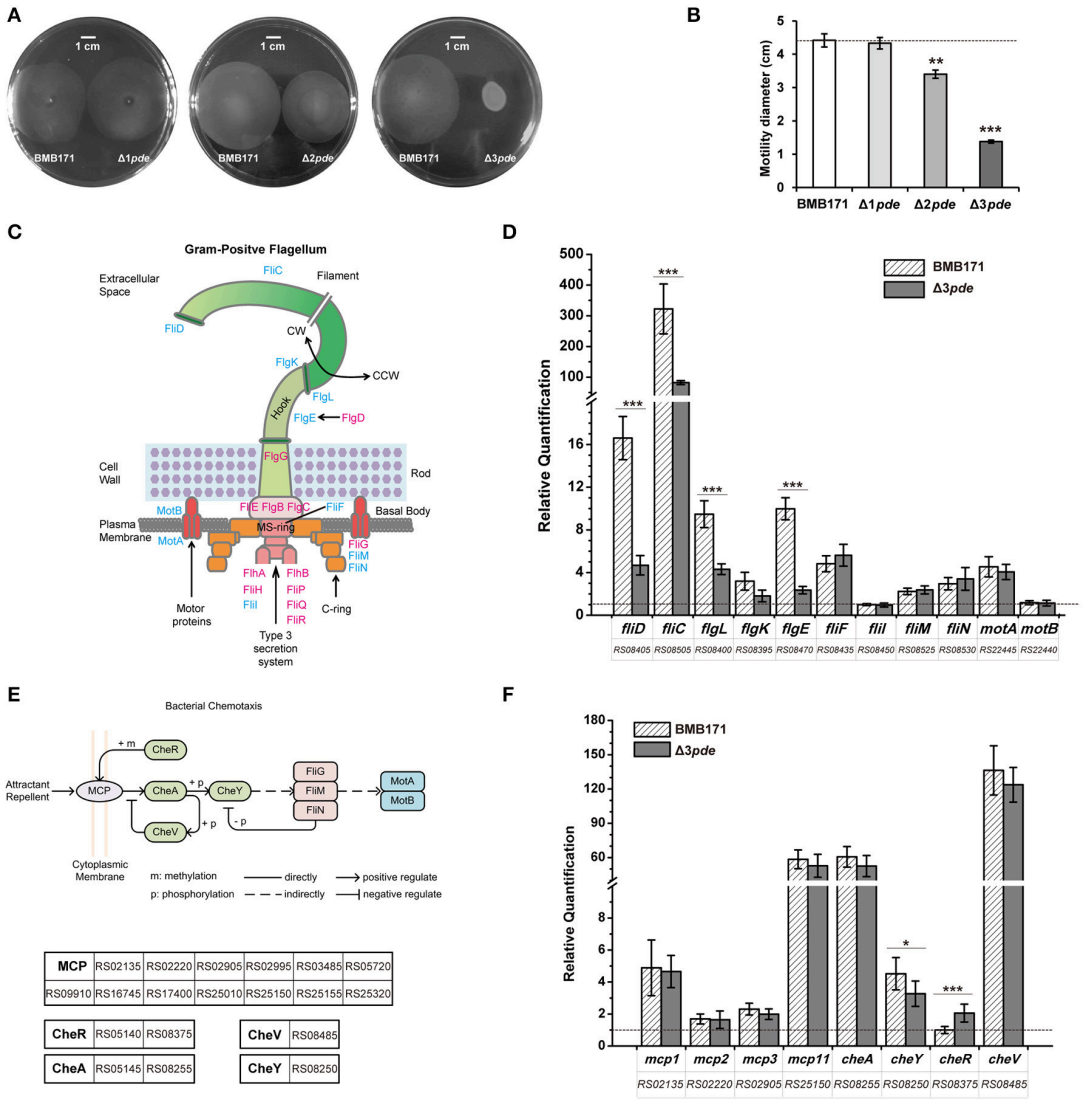
Cell motility of *B. thuringiensis* is repressed under elevated intracellular c-di-GMP level. **(A)** Representative motility plates of Δ1*pde* and BMB171 (left), Δ2*pde* and BMB171 (middle), and Δ3*pde* and BMB171 (right). Scale bar represents one centimeter. **(B)** Quantitative measurements of motilities in strains BMB171, Δ1*pde*, Δ2*pde*, and Δ3*pde* based on the colony diameter. Each experiment was repeated three times. The dash line represents the threshold of comparison in phenotype change. **(C)** Scheme for BMB171 bacterial flagellum (Schuhmacher et al., 2015). The major building blocks are color-coded as: basal body (pink and yellow), rod (pale green), hook (light green), and the filament (green). The flagellum can rotate either clockwise (CW) or counterclockwise (CCW). The detected genes were marked by blue. **(D)** Comparison of relative transcription levels of 11 genes related to flagellar assembly between Δ3*pde* and BMB171 using RT-qPCR. The reference gene is *gapdh* (glyceraldehyde 3-phosphate dehydrogenase) (Løvdal and Saha, 2014) for the RT-qPCR. **(E)** The regulatory pathway of bacterial chemotaxis system from BMB171. **(F)** Comparison of relative quantification in transcription level of eight genes involved in bacterial chemotaxis between Δ3*pde* and BMB171 using RT-qPCR. The dash line represents relative quantification that is equal to one. The two schemes for BMB171 were based on the KEGG PATHWAY Database (http://www.genome.jp/kegg-bin/show_pathway?btb02040). All the RT-qPCR data were average of three biological replicates (error bars are S.E. from mean value). The statistically significant differences were tested *via* a two-tailed *t*-test (^*^P ≤ 0.05, ^**^P ≤ 0.01, ^***^P ≤ 0.001).

Previous works on *B. subtilis, M. smegmatis*, and *C. difficile* have shown that c-di-GMP signaling is associated with cellular motility, due to the alteration of gene expression correlated with the flagellum assembly (Chen et al., 2012; Purcell et al., 2012; Gao et al., 2014; Gupta et al., 2015). We thus wonder whether change of intracellular c-di-GMP level influences the motility of *B. thuringiensis* as well. We examined the Δ1*pde*, Δ2*pde*, and Δ3*pde* mutants to check their cell motility on 0.5% LB agar plates at mid-exponential phase (9 h) (Figure 4A). The diameters of the swimming zones for Δ1*pde*, Δ2*pde*, and Δ3*pde* diminished gradually and displayed a dosage dependence effect (Figures 4A,B). The effect is especially obvious for Δ3*pde*, which showed a very small diameter compared to that of BMB171. In order to further explain this phenomenon, we searched 19 cell motility-associated genes *via* KEGG PATHWAY Databases (http://www.kegg.jp/kegg/pathway.html) (Figures 4C,E) and examined their gene expression changes by RT-qPCR (Figures 4D,F). Interestingly, the four genes *fliD, fliC, flgL*, and *flgE*, which are responsible for the hook and filament assembly, were found to be significantly down-regulated in Δ3*pde* compared to the parent strain BMB171 at transcription level (Figures 4C,D). The transcriptions of six genes responsible for the bottom part (*fliF, fliI, fliM*, and *fliN*) and rotor assembly of flagellum (*motA* and *motB*) did not exhibit much change in Δ3*pde* (Figures 4C,D). Based on that, we wondered whether the change of flagella-related genes expression impacts the bacterial falgella structure, so we observed the bacterial flagella directly using transmission electron microscope (TEM) and found that the flagellum number of Δ3*pde* was obviously less than that of BMB171 (Figure S10). Moreover, since previous studies already addressed that genes related to bacterial chemotaxis influenced cell motility as well (Paul et al., 2010), we have also selected eight genes involved in bacterial chemotaxis (Figure 4E) to check whether their expression change between the Δ3*pde* and BMB171 strains by RT-qPCR (Figure 4F). Rather unexpectedly, most genes involved in bacterial chemotaxis did not reveal any statistically meaningful change following elevated intracellular c-di-GMP level. The possible explanation for this bizarre behavior is that bacterial chemotaxis is possibly stimulated by certain sort of chemotaxis factors but not by c-di-GMP. Taken together, these results indicated that an elevated intracellular c-di-GMP level can repress cell motility by impeding transcription of a part of flagellum assembly genes, which is consistent with the reports for some Gram-positive bacteria (Chen et al., 2012; Purcell et al., 2012; Gao et al., 2014; Gupta et al., 2015) and Gram-negative bacteria (Simm et al., 2004; Kuchma et al., 2007; Paul et al., 2010).

### High intracellular c-di-GMP level promotes biofilm formation and cell-cell aggregation in *B. thuringiensis*

**Figure 5:**
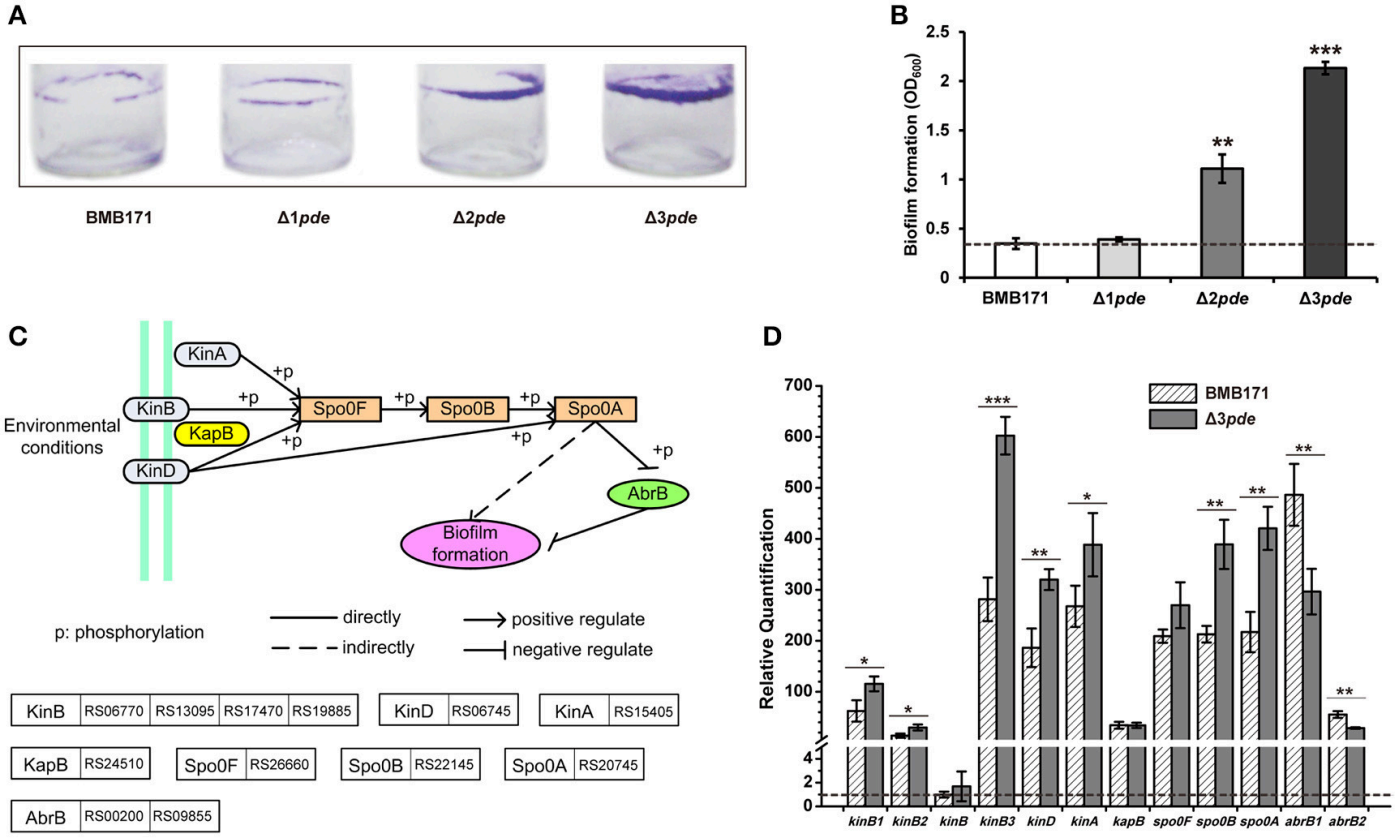
An increasing c-di-GMP level promotes biofilm formation and influences the transcription of relevant genes in *B. thuringiensis*. **(A)** Representative image of biofilm formation in BMB171, Δ1*pde*, Δ2*pde*, and Δ3*pde*. This assay was performed independently for at least three times. **(B)** Quantification of biofilm formation by optical density measurement. Error bar represents the standard deviation of three replicates. Dash line represents the criteria of comparison in phenotype change. **(C)** Scheme of potential biofilm formation pathway for BMB171. It was drawn based on the KEGG PATHWAY Database (http://www.kegg.jp/kegg-bin/show_pathway?btb02020). **(D)** Comparison of relative quantification in transcription level of 12 genes involved in biofilm formation between Δ3*pde* and BMB171 using RT-qPCR. The means and standard deviations were obtained from three replicates. The statistically significant differences were tested *via* a two-tailed *t*-test (^*^P ≤ 0.05, ^**^P ≤ 0.01, ^***^P ≤ 0.001). Dash line represents relative quantification that is equal to one.

In many Gram-negative bacteria, intracellular c-di-GMP increase not only inhibits motility but also up-regulates expression of factors that contribute to biofilm formation (Sondermann et al., 2012; Ha and O'Toole, 2015; Matsuyama et al., 2016). Thus, we also sought for biofilm formation change in response to increasing intracellular c-di-GMP level in *B. thuringiensis* by using a previously published method (Bordeleau et al., 2011). From the data, one can see that the biofilm production for both Δ2*pde* and Δ3*pde* were notably increased following increasing intracellular c-di-GMP level as expected, but the single *pde* mutant Δ1*pde* did not exhibit much change in the biofilm phenotype (Figures 5A,B). Additionally, we harvested cells of the Δ3*pde* and BMB171 samples during biofilm formation to determine the alteration in transcription level of the relevant genes, including those linked to sporulation and two-component system pathway leading to biofilm formation (Figure 5C), as well as to the global transcription regulator AbrB that has been reported to repress the biofilm formation in *B. subtilis* (Weng et al., 2013; Mielich-Süss and Lopez, 2015). During biofilm formation, all genes facilitating biofilm formation were mostly up-regulated *via* RT-qPCR detection, and the AbrB genes suppressing biofilm production were down-regulated as shown in Figure 5D. These results suggest that high c-di-GMP level did expedite biofilm formation in *B. thuringiensis*.

**Figure 6:**
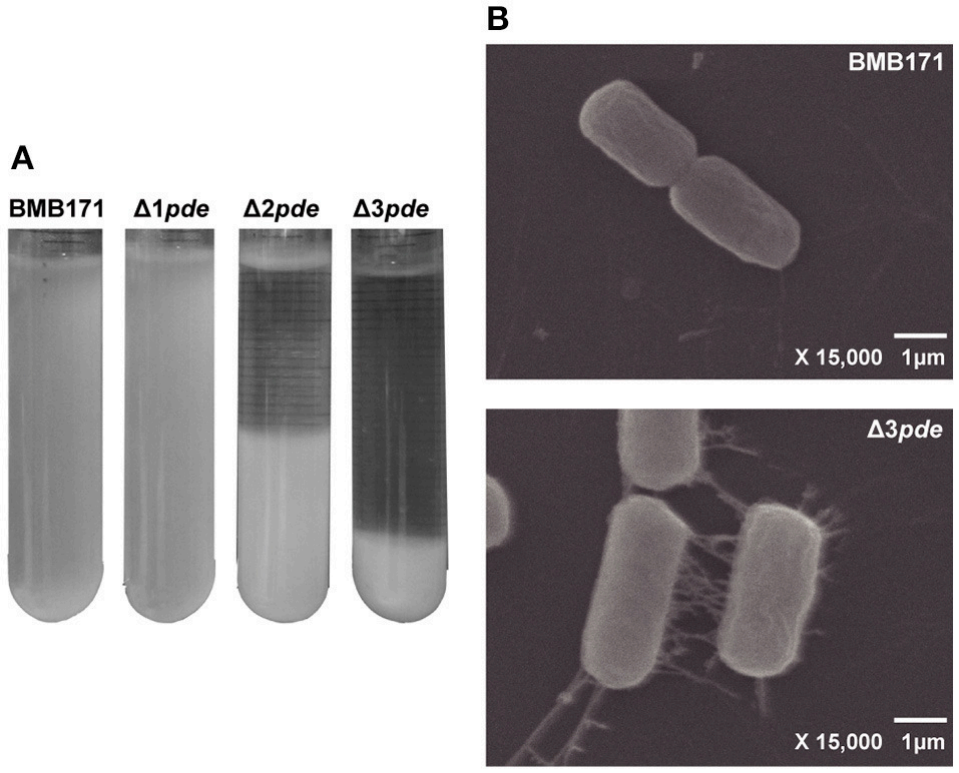
Degree of cell-cell aggregation is linked to the increasing intracellular c-di-GMP level in *B. thuringiensis*. **(A)** Comparison of cell-cell aggregation rate in BMB171, Δ1*pde*, Δ2*pde*, and Δ3*pde*. **(B)** SEM photograph (× 15,000) of BMB171 and Δ3*pde* during cell-cell aggregation assay. Many fibers were found to interconnect the cells in the Δ3*pde* mutant.

As biofilm formation assay were statically cultivated at room temperature for 6–8 h, we have noticed another phenotype change between BMB171 and *pde* gene deletion mutants, namely, the change in rate of cell-cell aggregation. This was also a dosage-dependent phenomenon observed during cell-cell aggregation among the Δ1*pde*, Δ2*pde*, and Δ3*pde* mutants as revealed in Figure 6A. Obviously, both Δ2*pde* and Δ3*pde* exhibited marked phenomenon of aggregation and settlement compared to Δ1*pde* and BMB171, thereby we picked the Δ3*pde* mutant with the clearest sediment difference and the parent strain BMB171 as control to further analyze this phenotype alteration by using the scanning electron microscope (SEM). As shown in Figure 6B, the cells of Δ3*pde* were connected together by an extensive fibrous network, while that of the control strain BMB171 were completely devoid of such fiber connection when cells were grown under the same conditions. Unexpectedly, the control cells of BMB171 could hardly grow in group on the tissue culture-treated slide, so that we only observed a few cells on an entire cell slide. Therefore, we concluded that this extensive fibrous network is probable a key factor in cell growth adherent to this abiotic surface.

### High intracellular c-di-GMP level plays a key role in the insecticidal activity of *B. thuringiensis* against cotton bollworm larva

**Figure 7:**
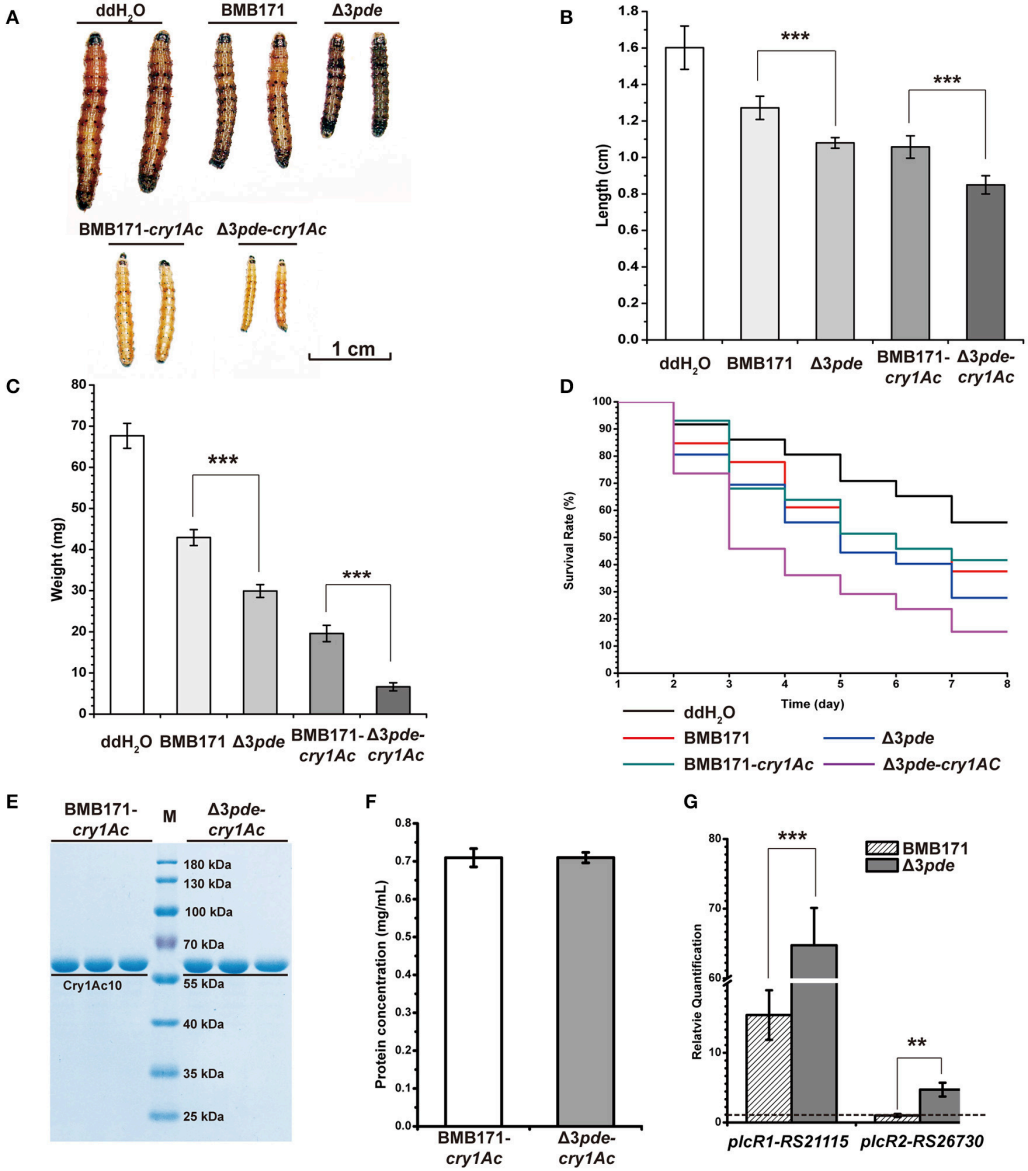
An increasing intracellular c-di-GMP level enhances the insecticidal activity of Δ3*pde* and Δ3*pde*-*cry1Ac* at the late stationary phase or the early sporulation phase (48 h). The strain pairs to be assayed were BMB171, Δ3*pde* and BMB171*-cry1Ac*, Δ3*pde*-*cry1Ac*, with ddH_2_O as a negative control. Both BMB171 and Δ3*pde* strains were applied using the original concentrations, but BMB171*-cry1Ac* and Δ3*pde*-*cry1Ac* strains were diluted 1000-fold before applying. **(A)** The image of cotton bollworm larvae fed with different strains with different degrees of dilution at the end of rearing cycle. Scale bar represents 1 cm. **(B)** Quantitative measurements in length of cotton bollworm larvae fed with these different strains with different degrees of dilution. **(C)** Quantitative measurements in weight of cotton bollworm larvae fed with these different strains with different degrees of dilution. **(D)** Comparison in survival rate of cotton bollworm larvae fed with different stains with different degrees of dilution within rearing cycle (7 day). **(E)** Concentrations of Cry1Ac in BMB171-*cry1Ac* and Δ3*pde*-*cry1Ac* at 48 h analyzed by SDS-PAGE. **(F)** Concentrations of Cry1Ac at 48 h determined by Bradford method. **(G)** Comparison of relative transcription levels of *plcR1* and *plcR2* genes between BMB171 and Δ3*pde* using RT-qPCR method. Error bars represent the standard deviation of three replicates. The statistically meaningful differences were tested *via* a two-tailed *t*-test (^*^P ≤ 0.05, ^**^P ≤ 0.01, ^***^P ≤ 0.001). Dash line represents relative quantification that is equal to one.

Although BMB171 cannot produce ICPs because it is an acrystalliferous mutant generated from the wild-type crystalliferous strain YBT-1463 (Li et al., 2000; He et al., 2010), it is still virulent to certain kinds of insects for possessing other exogenous virulence factors including enterotoxins, hemolysins, phospholipases and proteases and so on (Bravo et al., 2011; Wang et al., 2013b). In order to rigorously explore the virulence change in response to altering intracellular c-di-GMP levels in BMB171, we have constructed two recombinant strains BMB171-*cry1Ac* and Δ3*pde*-*cry1Ac*, which harbored authentic gene for the parasporal crystal Cry1Ac10 production. We selected *Helicoverpa armigera* as the host insect and fed them using the four individual strains of *B. thuringiensis*, namely, BMB171, Δ3*pde*, BMB171-*cry1Ac* and Δ3*pde*-*cry1Ac*, respectively. Both BMB171 and Δ3*pde* strains were applied using the original concentrations, but the BMB171*-cry1Ac* and Δ3*pde*-*cry1Ac* strains were diluted 1000-fold before applying. We then estimated the virulence of these four strains against *H. armigera* at the late stationary phase or the early sporulation phase (48 h) (Figure S2) by using the following three important criteria: (1) the survival rate of cotton bollworm larvae under certain concentration of bacterial strain within a raising cycle; (2) the length; and (3) the weight of cotton bollworm larvae at the end of feeding cycle. Based on the bioassay results, the cotton bollworm larvae fed with BMB171 and BMB171-*cry1Ac* were found to grow significantly better than those fed with Δ3*pde* and Δ3*pde*-*cry1Ac*, respectively (Figure 7A). There were also striking length decrease (Figure 7B) and weight loss (Figure 7C) for cotton bollworm larvae fed with Δ3*pde* and Δ3*pde*-*cry1Ac* compared to BMB171 and BMB171-*cry1Ac*. Importantly, the survival rate of cotton bollworm larvae fed with strain Δ3*pde* and Δ3*pde*-*cry1Ac* obviously decreased compared to those fed with the BMB171 and BMB171-*cry1Ac* (Figure 7D). To understand the cause of these differences, we also determined concentration of Cry1Ac10 protein in the BMB171-*cry1Ac* and Δ3*pde*-*cry1A* strains through SDS-PAGE and Bradford method, but found that there is not much change in the concentration of Cry1Ac10 protein (Figures 7E,F) in the two strains. This result indicated that the c-di-GMP does not affect the expression of endogenous virulence factor Cry1Ac10. Therefore, we supposed that c-di-GMP might change the expression of other exogenous virulence factors. To verify this speculation, we further checked transcription level changes between BMB171 and Δ3*pde* of the gene *plcR*, which encodes a pleiotropic regulator for extracellular virulence factor gene expression in *B. thuringiensis* (Kim et al., 2015; Slamti et al., 2016). Indeed, the transcription level changes of *plcR* genes (*plcR1*:*RS21115* and *plcR2*:*RS26730*) in Δ3*pde* increased by 3.2- and 3.7-fold compared to those in BMB171, respectively (Figure 7G). Taken together, these data demonstrate that high level of c-di-GMP affected the expression of *plcR* genes and had a role in the insecticidal activity of *B. thuringiensis* against cotton bollworm larvae *in vivo*. On the contrary, when the transcription level of the *plcR* genes in Δ3*pde* and BMB171 were determined at the mid exponential phase (9 h), the virulence of Δ3*pde* showed little change to that of BMB171 (Figure S10). It may be due to the fact that a large proportion of the virulence genes had not yet been induced at the mid exponential phase (Wang et al., 2013a,b).

### Effect of high c-di-GMP level on transcription of *B. thuringiensis* in genome-scale

The abundance and tempo-spatial distribution of bacterial proteins involved in c-di-GMP metabolism indicate the significance of c-di-GMP signaling pathways in bacteria (Chen et al., 2012; Purcell et al., 2012; Gao et al., 2014; Gupta et al., 2015). However, little is known with respect to the influence of intracellular c-di-GMP level on transcription profiles in Gram-positive bacteria compared to Gram-negative bacteria (Beyhan et al., 2006; Mendez-Ortiz et al., 2006; Ueda and Wood, 2009; Ferreira et al., 2013; An et al., 2014; MacKenzie et al., 2015). Therefore, we performed a comprehensive RNA-seq approach to investigate the effect of c-di-GMP on transcriptionin genome-scale, hopefully to be able to discover novel regulatory pathways mediated by c-di-GMP in the Gram-positive *B. thuringiensis*. In this regard, we collected their RNA samples when Δ3*pde* and BMB171 were grown at mid-exponential phase (9 h, see Figure S2), and performed RNA-seq to check their expression levels. The completed 5.64-Mb genome of the BMB171 was used as a reference, which contains a circular chromosome (5.33 Mb) containing 5,088 predicted open reading frames (ORFs) and a circular plasmid pBMB171 (0.31 Mb encoding 276 predicted ORFs (He et al., 2010). Using filtering conditions of larger than 1.5-fold change in gene expression (Neusser et al., 2010) and of false discovery rate (FDR) (Benjamini and Hochberg, 1995) less than 0.001 as criteria to judge the significance of gene transcription difference, we identified 233 differentially transcribed genes (4.3% of total), including 132 repressed genes and 101 enhanced genes in the Δ3*pde* strain compared to the control strain BMB171. The complete list of differently transcribed genes is shown in the Supplementary Material (Table S4).

**Figure 8:**
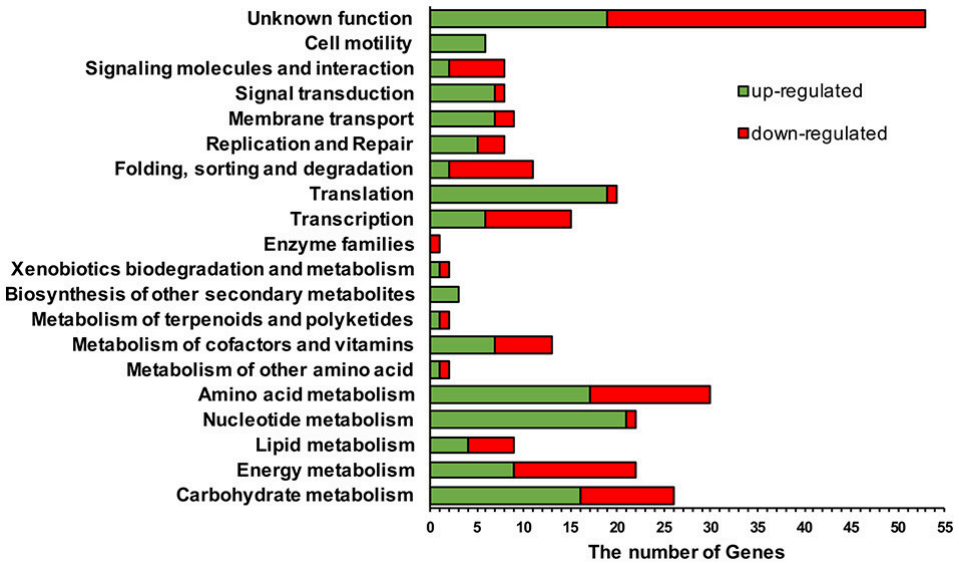
Functional categories of differentially expressed genes in response to an increasing intracellular c-di-GMP level. The number of genes that are up-regulated (red) or repressed (green) in response to increasing c-di-GMP level is presented according to the functions assigned for them by the KOBAS 2.0 and their functional annotation.

Based on the analysis results of KOBAS (KEGG Orthology Based Annotation System) 3.0 program (http://kobas.cbi.pku.edu.cn) and annotated functions provided by the *B. thuringiensis* genome sequencing project, the genes affected by high c-di-GMP level are predicted to involve in various cellular functions (Figure 8). There were eight regulatory pathways with more than ten genes influenced by high c-di-GMP level. Notably, in mutant Δ3*pde*, there were 40 genes down-regulated and only two genes up-regulated involved in nucleotide metabolism and translation pathway (Table S5). In other words, genes encoding ribosome family proteins and purine or pyrimidine metabolism proteins were repressed following an artificially elevated intracellular c-di-GMP level. In addition, we found that 22.8% (53 out of 233) (Figure 8) of the genes that were differentially regulated in response to increased c-di-GMP concentration are predicted to encode many hypothetical or conserved hypothetical proteins. These genes are organized into 53 different transcripts and 96.2% of them are located on the chromosome (Table S4). Identification of such a large set of hypothetical proteins regulated by c-di-GMP indicates that we in fact know little on the c-di-GMP-regulated physiology and behavior. It is of particular interest to note that one of these genes, *RS18010*, which is located at the same operon as *RS18005* (encoding an acetyltransferase), encodes a hypothetical protein whose transcription level was very high and increased by 1.54-fold in Δ3*pde* (RPKM of 75480 vs. 49004, see Table S4). Moreover, through our transcriptome data, we further confirmed the result of cell motility between BMB171 and Δ3*pde* (Figure 4 and Table S5). These results suggest these regulatory pathways are under direct or indirect influence of c-di-GMP.

## Discussion

### Some putative proteins with GGDEF and/or EAL domains in *B. thuringiensis* may be inactive

In this study, enzyme activity assays indicated that the putative proteins with GGDEF and/or EAL domains exhibit substantial differences in controlling c-di-GMP metabolism. For example, RS19795 with only a single EAL domain, and RS02850, RS03240, RS17435, and RS18570 containing both EAL and GGDEF domains, all exhibited considerable PDE activity *in vitro*. However, both RS19835 and RS26115, which were predicted with a GGDEF domain by Pfam database 28.0, were in fact not identified to harbor a GGDEF domain by SMART database. The reason why they were annotated as putative proteins with a GGDEF may be due to the presence of a degenerate GGDEF domain yet found to adopt a similar 3D structure as the conserved GGDEF domain.

Another interesting protein is RS26720 that contains a dual GGDEF-EAL domain but with no PDE (Figure 2H) or DGC activity (Figure S7I) *in vitro*. It is possible due to the fact that its N-terminal transmembrane domain needed to be truncated to make it soluble for the *in vitro* study. Deletion of the transmembrane domain also removed the PAS domain (Zhulin et al., 1997; Taylor and Zhulin, 1999) and PAC domain that may play a crucial role in dimerizing the GGDEF domain important for DGC activity. Such a high diversity of the N-terminal domains implies that their functions are not redundant. These multi-domain c-di-GMP-metabolizing enzymes are possibly implicated individually in different temporal or spatial regulatory pathways (Sondermann et al., 2012; Zheng et al., 2015). In addition, RS19835 and RS26115 were both found to be inactive (Figures S7C,D), possibly due to the fact that their GGDEF domains are degenerated. But each could serve as a receptor of c-di-GMP in certain c-di-GMP signaling pathway (Chou and Galperin, 2016).

### The c-di-GMP level of *B. thuringiensis* is low but exhibits significant impacts on it's physiology and phenotype

After examining the intracellular c-di-GMP level in the Gram-positive *B. thuringiensis* BMB171, we observed a lower level of c-di-GMP at the stationary phase than at the exponential phase in both Δ3*pde* and BMB171 strains. Regarding the c-di-GMP turnover, the number of active PDEs is more than that of active DGCs, meaning that the c-di-GMP concentration in *B. thuringiensis* is probably at a lower level. Or speculatively, c-di-GMP secretion pathway may be present. However, the intracellular c-di-GMP concentration in the Gram-positive *B. thuringiensis* was considerably lower beyond our expectation, which is two orders of magnitudes lower than that of c-di-AMP (Zheng et al., 2015). Yet, amazingly, even at such a low level, c-di-GMP still exhibited considerable effects on a wide range of phenotypes of *B. thuringiensis*, such as cell motility, biofilm formation, cell-cell aggregation and insecticidal activity for cotton bollworm larvae.

### The altered c-di-GMP-regulated phenotypes implicate the great potential of *Bx. thuringiensis* for novel application

The current study showed that the insecticidal activity of *B. thuringiensis* against cotton bollworm larvae increased with increasing c-di-GMP level. Considering the diversity and complexity of different virulence systems in a wide variety of pathogenic bacteria (Raymond et al., 2010; Bravo et al., 2011), we supposed that the virulence is interconnected or interdependent with other cell morphology phenotypes including cell motility, biofilm formation and cell-cell aggregation (Sondermann et al., 2012). In addition, increasing biofilm formation regulated by a high intracellular c-di-GMP level may promote resistance of *B. thuringiensis* to a wide variety of adverse environments, which is advantageous for the adaptability of *B. thuringiensis* as a living insecticide. Although the entire regulatory pathway mediated by c-di-GMP is still unclear in *B. thuringiensis*, our construction of Δ3*pde* offers a new strategy for improving insecticidal activity and subsequent applications.

### Potential crosstalk between c-di-GMP and c-di-AMP in *B. thuringiensis*

c-di-AMP is another important cyclic-di-nucleotide second messenger molecule, which regulate various cellular processes including sporulation, cell wall homeostasis, potassium ion transport, virulence and host immune activation (Woodward et al., 2010; Corrigan and Gründling, 2013; Römling et al., 2013; Zheng et al., 2015; He et al., 2016; Cai and He, 2017; Whiteley et al., 2017). The GdpP-like proteins belong to the DHH family, which can hydrolyze a wide range of substrates from pyrophosphate to ssDNA, and can degrade both c-di-AMP and c-di-GMP such as YybT in *B. subtilis* (Rao et al., 2010; Wang et al., 2017), GdpP in *Staphylococcus aureus* (Corrigan et al., 2011), MSMEG_2630 in *M. smegmatis* (Tang et al., 2015) or Rv2837c in *M. tuberculosis* (He et al., 2016).

According to the UniPort database, YybT (BMB171_RS27370), YtqI (BMB171_RS22925), and RecJ (BMB171_RS21990) in *B. thuringiensis* belongs to DHH family proteins, however, our result (data not shown) and previous reports showed that both YtqI and RecJ are not c-di-AMP-specific PDEs (Mechold et al., 2007). Only YybT in *B. thuringiensis* has 62% identities in the DHH and GGGH residues compared to that in *B. subtilis* (Figure S12) and can not only hydrolyze c-di-AMP to 5′-pApA, but also hydrolyze c-di-GMP to 5′-pGpG, yet the difference in Michaelis-Menten constant (*K*_*m*_) between c-di-AMP and c-di-GMP is two orders of magnitude (data not shown). The result is consistent with the other studies (Rao et al., 2010; Corrigan et al., 2011; Tang et al., 2015; He et al., 2016), indicating that YybT prefers c-di-AMP over c-di-GMP as a physiological substrate (Tang et al., 2015). It is the only connection point between the c-di-AMP and c-di-GMP regular pathways at the metabolic enzyme levels. Whether there are common and crossed targets for the two cyclic-di-nucleotide messenger molecules in *B. thuringiensis* is worth studying further.

### Regulatory network mediated by c-di-GMP in *B. thuringiensis*

Based on our previous and current studies, we have delineated a partial regulatory network mediated by c-di-GMP in *B. thuringiensis* (Kalia et al., 2013; Mielich-Süss and Lopez, 2015; Slamti et al., 2016). We propose that c-di-GMP is an important regulator which suppresses cell motility by decreasing the transcription level of genes related to flagellum assembly (Figure 4 and Table S5), promotes biofilm information by repressing the expression of the transcription factor AbrB, and may intensify the insecticidal activity by up-regulating the transcription of the transcription factor PlcR. However, the integrated regulatory pathway between c-di-GMP and the above described physiological activities remains largely unknown. To solidify this connection, we are currently looking for receptor or effector proteins that can directly bind c-di-GMP in *B. thuringiensis*. The c-di-GMP concentration is very low in *B. thuringiensis*, but still exhibits significant phenotypes, meaning that there exist specific c-di-GMP receptors with strong affinity for c-di-GMP. In *B. subtilis*, there is a c-di-GMP receptor protein DgrA with a PilZ domain as reported by Gao et al. (2013). But in *B. thuringiensis*, we did not find any proteins with the PilZ domain. Similarly, we could not seek out any protein with a considerable homology with known protein receptors. Thus, those proteins with degenerate GGDEF or EAL domain are worthy of examination for their binding with c-di-GMP in the future study. On the other hand, two riboswitches, termed as Bc1 RNA and Bc2 RNA, are located in the 5′-UTR of a *mcp* RNA encoding for methyl-accepting chemotaxis protein and in the 5′-UTR of a *cap* RNA encoding for collagen adhesion protein (Tang et al., 2016), respectively.

Moreover, through RNA-seq analysis, many other regulation pathways such as the translation and nucleotide metabolism and many unknown function proteins are found to be highly affected by c-di-GMP concentration. Those proteins with unknown function are likely important targets to link phenotype with c-di-GMP for leading to a more complete c-di-GMP signaling regulatory network (Beyhan et al., 2006). The regulatory mechanisms mediated by c-di-GMP in *B. thuringiensis* are now undergoing in our laboratory.

## Author contributions

YF and JH conceived and designed the study. YF completed most of the bioinformatics analysis. YF, ZY, SL, and BC performed most of the experiments and made most of the data evaluation. ZL and LZ participated in partial experiments. YF, S-HC, and JH contributed to the writing of the manuscript. All listed authors had read and approved the final manuscript version.

### Conflict of interest statement

The authors declare that the research was conducted in the absence of any commercial or financial relationships that could be construed as a potential conflict of interest.

## References

[B1] Abel S. Chien P. Wassmann P. Schirmer T. Kaever V. Laub M. T.. (2011). Regulatory cohesion of cell cycle and cell differentiation through interlinked phosphorylation and second messenger networks. Mol. Cell 43, 550–560. 10.1016/j.molcel.2011.07.018 21855795 PMC3298681

[B2] Albert-Weissenberger C. Sahr T. Sismeiro O. Hacker J. Heuner K. Buchrieser C. (2010). Control of flagellar gene regulation in *Legionella pneumophila* and its relation to growth phase. J. Bacteriol. 192, 446–455. 10.1128/JB.00610-09 19915024 PMC2805308

[B3] Aldridge P. Paul R. Goymer P. Rainey P. Jenal U. (2003). Role of the GGDEF regulator PleD in polar development of *Caulobacter crescentus*. Mol. Microbiol. 47, 1695–1708. 10.1046/j.1365-2958.2003.03401.x 12622822

[B4] Amikam D. Galperin M. (2006). PilZ domain is part of the bacterial c-di-GMP binding protein. Bioinformatics 22, 3–9. 10.1093/bioinformatics/bti739 16249258

[B5] An S. Q. Caly D. L. McCarthy Y. Murdoch S. L. Ward J. Febrer M.. (2014). Novel cyclic di-GMP effectors of the YajQ protein family control bacterial virulence. PLoS Pathog. 10:e1004429. 10.1371/journal.ppat.1004429 25329577 PMC4199771

[B6] Aragón I. M. Pérez-Mendoza D. Gallegos M. T. Ramos C. (2015). The c-di-GMP phosphodiesterase BifA is involved in the virulence of bacteria from the *Pseudomonas syringae* complex. Mol. Plant Pathol. 16, 604–615. 10.1111/mpp.12218 25385023 PMC6638514

[B7] Ausmees N. Mayer R. Weinhouse H. Volman G. Amikam D. Benziman M.. (2001). Genetic data indicate that proteins containing the GGDEF domain possess diguanylate cyclase activity. FEMS Microbiol. Lett. 204, 163–167. 10.1111/j.1574-6968.2001.tb10880.x 11682196

[B8] Benjamini Y. Hochberg Y. (1995). Controlling the false discovery rate: a practical and powerful approach to multiple testing. J. R. Stat. Soc. Ser. B 57, 289–300.

[B9] Beyhan S. Odell L. S. Yildiz F. H. (2008). Identification and characterization of cyclic diguanylate signaling systems controlling rugosity in *Vibrio cholerae*. J. Bacteriol. 190, 7392–7405. 10.1128/JB.00564-08 18790873 PMC2576663

[B10] Beyhan S. Tischler A. D. Camilli A. Yildiz F. H. (2006). Transcriptome and phenotypic responses of *Vibrio cholerae* to increased cyclic di-GMP level. J. Bacteriol. 188, 3600–3613. 10.1128/JB.188.10.3600-3613.2006 16672614 PMC1482859

[B11] Bharati B. K. Sharma I. M. Kasetty S. Kumar M. Mukherjee R. Chatterji D. (2012). A full-length bifunctional protein involved in c-di-GMP turnover is required for long-term survival under nutrient starvation in *Mycobacterium smegmatis*. Microbiology (Reading. Engl). 158, 1415–1427. 10.1099/mic.0.053892-0 22343354

[B12] Boehm A. Kaiser M. Li H. Spangler C. Kasper C. Ackermann M.. (2010). Second messenger-mediated adjustment of bacterial swimming velocity. Cell 141, 107–116. 10.1016/j.cell.2010.01.018 20303158

[B13] Bordeleau E. Fortier L. C. Malouin F. Burrus V. (2011). c-di-GMP turn-over in *Clostridium difficile* is controlled by a plethora of diguanylate cyclases and phosphoesterases. PLoS Genet. 7:e1002039 10.1371/journal.pgen.1002039 21483756 PMC3069119

[B14] Bordeleau E. Purcell E. B. Lafontaine D. A. Fortier L. C. Tamayo R. Burrus V. (2015). Cyclic di-GMP riboswitch-regulated type IV pili contribute to aggregation of *Clostridium difficile*. J. Bacteriol. 197, 819–832. 10.1128/JB.02340-14 25512308 PMC4325102

[B15] Bravo A. Likitvivatanavong S. Gill S. S. Soberón M. (2011). *Bacillus thuringiensis*: a story of a successful bioinsecticide. Insect Biochem. Mol. Biol. 41, 423–431. 10.1016/j.ibmb.2011.02.006 21376122 PMC3689885

[B16] Cai X. He J. (2017). Second messenger c-di-AMP regulates potassium ion transport. Wei Sheng Wu Xue Bao 57, 1434–1442. 10.13343/j.cnki.wsxb.20160479

[B17] Chan C. Paul R. Samoray D. Amiot N. C. Giese B. Jenal U.. (2004). Structural basis of activity and allosteric control of diguanylate cyclase. Proc. Natl. Acad. Sci. U.S.A. 101, 17084–17089. 10.1073/pnas.0406134101 15569936 PMC535365

[B18] Chen Y. Chai Y. Guo J. H. Losick R. (2012). Evidence for cyclic di-GMP-mediated signaling in *Bacillus subtilis*. J. Bacteriol. 194, 5080–5090. 10.1128/JB.01092-12 22821967 PMC3430322

[B19] Chen Y. Liu S. Liu C. Huang Y. Chi K. Su T.. (2016). Dcsbis (PA2771) from *Pseudomonas aeruginosa* is a highly active diguanylate cyclase with unique activity regulation. Sci. Rep. 6:29499. 10.1038/srep29499 27388857 PMC4937426

[B20] Chen Z. H. Schaap P. (2016). Secreted cyclic di-GMP induces stalk cell differentiation in the eukaryote *Dictyostelium discoideum*. J. Bacteriol. 198, 27–31. 10.1128/JB.00321-15 26013485 PMC4686194

[B21] Chin K. H. Lee Y. C. Tu Z. L. Chen C. H. Tseng Y. H. Yang J. M.. (2010). The cAMP receptor-like protein CLP is a novel c-di-GMP receptor linking cell-cell signaling to virulence gene expression in *Xanthomonas campestris*. J. Mol. Biol. 396, 646–662. 10.1016/j.jmb.2009.11.076 20004667

[B22] Chou S. H. Galperin M. Y. (2016). Diversity of c-di-GMP-binding proteins and mechanisms. J. Bacteriol. 198, 32–46. 10.1128/JB.00333-15 26055114 PMC4686193

[B23] Christen M. Christen B. Folcher M. Schauerte A. Jenal U. (2005). Identification and characterization of a cyclic di-GMP-specific phosphodiesterase and its allosteric control by GTP. J. Biol. Chem. 280, 30829–30837. 10.1074/jbc.M504429200 15994307

[B24] Corrigan R. M. Abbott J. C. Burhenne H. Kaever V. Gründling A. (2011). c-di-AMP is a new second messenger in *Staphylococcus aureus* with a role in controlling cell size and envelope stress. PLoS Pathog. 7:e1002217. 10.1371/journal.ppat.1002217 21909268 PMC3164647

[B25] Corrigan R. M. Gründling A. (2013). Cyclic di-AMP: another second messenger enters the fray. Nat. Rev. Microbiol. 11, 513–524. 10.1038/nrmicro3069 23812326

[B26] Deng C. Peng Q. Song F. Lereclus D. (2014). Regulation of cry gene expression in *Bacillus thuringiensis*. Toxins (Basel). 6, 2194–2209. 10.3390/toxins6072194 25055802 PMC4113751

[B27] Ferreira A. S. Silva I. N. Oliveira V. H. Becker J. D. Givskov M. Ryan R. P.. (2013). Comparative transcriptomic analysis of the *Burkholderia cepacia* tyrosine kinase BceF mutant reveals a role in tolerance to stress, biofilm formation, and virulence. Appl. Environ. Microbiol. 79, 3009–3020. 10.1128/AEM.00222-13 23435894 PMC3623129

[B28] Finn R. D. Bateman A. Clements J. Coggill P. Eberhardt R. Y. Eddy S. R.. (2014). Pfam: the protein families database. Nucleic Acids Res. 42, D222–D230. 10.1093/nar/gkt1223 24288371 PMC3965110

[B29] Gao X. Dong X. Subramanian S. Matthews P. M. Cooper C. A. Kearns D. B.. (2014). Engineering of *Bacillus subtilis* strains to allow rapid characterization of heterologous diguanylate cyclases and phosphodiesterases. Appl. Environ. Microbiol. 80, 6167–6174. 10.1128/AEM.01638-14 25085482 PMC4178680

[B30] Gao X. Mukherjee S. Matthews P. M. Hammad L. A. Kearns D. B. Dann C. E.. (2013). Functional characterization of core components of the *Bacillus subtilis* cyclic-di-GMP signaling pathway. J. Bacteriol. 195, 4782–4792. 10.1128/JB.00373-13 23893111 PMC3807487

[B31] Gupta K. R. Kasetty S. Chatterji D. (2015). Novel functions of (p)ppGpp and cyclic di-GMP in mycobacterial physiology revealed by phenotype microarray analysis of wild-type and isogenic strains of *Mycobacterium smegmatis*. Appl. Environ. Microbiol. 81, 2571–2578. 10.1128/AEM.03999-14 25636840 PMC4357947

[B32] Ha D. G. O'Toole G. A. (2015). c-di-GMP and its effects on biofilm formation and dispersion: a *Pseudomonas aeruginosa* review. Microbiol. Spectr. 3, MB-0003–2014. 10.1128/microbiolspec.MB-0003-2014 26104694 PMC4498269

[B33] He J. Shao X. Zheng H. Li M. Wang J. Zhang Q.. (2010). Complete genome sequence of *Bacillus thuringiensis* mutant strain BMB171. J. Bacteriol. 192, 4074–4075. 10.1128/JB.00562-10 20525827 PMC2916366

[B34] He J. Wang J. Yin W. Shao X. Zheng H. Li M.. (2011). Complete genome sequence of *Bacillus thuringiensis* subsp. *chinensis* strain CT-43. J. Bacteriol. 193, 3407–3408. 10.1128/JB.05085-11 21551307 PMC3133296

[B35] He Q. Wang F. Liu S. Zhu D. Cong H. Gao F. (2016). Structural and biochemical insight into the mechanism of Rv2837c from *Mycobacterium tuberculosis* as a c-di-NMP phosphodiesterase. J. Biol. Chem. 291, 3668–3681. 10.1074/jbc.M115.699801 26668313 PMC4751404

[B36] Janes B. K. Stibitz S. (2006). Routine markerless gene replacement in *Bacillus anthracis*. Infect. Immun. 74, 1949–1953. 10.1128/IAI.74.3.1949-1953.2006 16495572 PMC1418658

[B37] Kalia D. Merey G. Nakayama S. Zheng Y. Zhou J. Luo Y. (2013). Nucleotide, c-di-GMP, c-di-AMP, cGMP, cAMP, (p)ppGpp signaling in bacteria and implications in pathogenesis. Chem. Soc. Rev. 47, 305–341. 10.1002/chin.201316274 23023210

[B38] Kariisa A. T. Grube A. Tamayo R. (2015). Two nucleotide second messengers regulate the production of the *Vibrio cholerae* colonization factor GbpA. BMC Microbiol. 15, 166. 10.1186/s12866-015-0506-5 26286031 PMC4545359

[B39] Kariisa A. T. Weeks K. Tamayo R. (2016). The RNA domain Vc1 regulates downstream gene expression in response to cyclic diguanylate in *Vibrio cholerae*. PLoS ONE 11:e0148478. 10.1371/journal.pone.0148478 26849223 PMC4744006

[B40] Kim M. J. Han J. K. Park J. S. Lee J. S. Lee S. H. Cho J. I.. (2015). Various enterotoxin and other virulence factor genes widespread among *Bacillus cereus* and *Bacillus thuringiensis* strains. J. Microbiol. Biotechnol. 25, 872–879. 10.4014/jmb.1502.02003 25791850

[B41] Krasteva P. V. Fong J. C. Shikuma N. J. Beyhan S. Navarro M. V. Yildiz F. H. (2010). *Vibrio cholera* VpsT regulates matrix production and motility by directly sensing cyclic di-GMP. Science 327, 866–868. 10.1126/science.1181185 20150502 PMC2828054

[B42] Kuchma S. L. Brothers K. M. Merritt J. H. Liberati N. T. Ausubel F. M. O'Toole G. A. (2007). BifA, a cyclic-di-GMP phosphodiesterase, inversely regulates biofilm formation and swarming motility by *Pseudomonas saeruginosa* PA14. J. Bacteriol. 189, 8165–8178. 10.1128/JB.00586-07 17586641 PMC2168662

[B43] Langmead B. Salzberg S. (2012). Fast gapped-read alignment with Bowtie 2. Nat. Methods 9, 357–359. 10.1038/nmeth.1923 22388286 PMC3322381

[B44] Lee V. T. Matewish J. M. Kessler J. L. Hyodo M. Hayakawa Y. Lory S. (2007). A cyclic-di-GMP receptor required for bacterial exopolysaccharide production. Mol. Microbiol. 65, 1474–1484. 10.1111/j.1365-2958.2007.05879.x 17824927 PMC2170427

[B45] Li L. Shao Z. Yu Z. (2000). Transformation of *Bacillus thuringiensis* recipient BMB171 by electroporation. Wei Sheng Wu Xue Tong Bao 27, 331–334.

[B46] Li X. Chen F. Xiao J. He J. (2017a). Structure and function of c-di-GMP riboswitches. Sheng Wu Gong Cheng Xue Bao 33, 1357–1368. 10.13345/j.cjb.170085 28956387

[B47] Li X. Mei H. Chen F. Tang Q. Yu Z. Cao X.. (2017b). Transcriptome landscape of *Mycobacterium smegmatis*. Front. Microbiol. 8:2505. 10.3389/fmicb.2017.02505 29326668 PMC5741613

[B48] Lori C. Ozaki S. Steiner S. Bohm R. Abel S. Dubey B. N.. (2015). Cyclic di-GMP acts as a cell cycle oscillator to drive chromosome replication. Nature 523, 236–239. 10.1038/nature14473 25945741

[B49] Løvdal T. Saha A. (2014). Reference gene selection in *Carnobacterium maltaromaticum, Lactobacillus curvatus*, and *Listeria innocua* subjected to temperature and salt stress. Mol. Biotechnol. 56, 210–222. 10.1007/s12033-013-9697-x 24037409

[B50] Lucena W. A. Pelegrini P. B. Martins-de-Sa D. Fonseca F. C. Gomes J. E. Jr. de Macedo L. L.. (2014). Molecular approaches to improve the insecticidal activity of *Bacillus thuringiensis* Cry toxins. Toxins (Basel) 6, 2393–2423. 10.3390/toxins6082393 25123558 PMC4147589

[B51] MacKenzie K. D. Wang Y. Shivak D. J. Wong C. S. Hoffman L. J. Lam S.. (2015). Bistable expression of CsgD in *Salmonella enterica* serovar Typhimurium connects virulence to persistence. Infect. Immun. 83, 2312–2326. 10.1128/IAI.00137-15 25824832 PMC4432751

[B52] Marchler-Bauer A. Derbyshire M. K. Gonzales N. R. Lu S. Chitsaz F. Geer L. Y.. (2015). CDD: NCBI's conserved domain database. Nucleic Acids Res. 43, D222–D226. 10.1093/nar/gku1221 25414356 PMC4383992

[B53] Matsuyama B. Y. Krasteva P. V. Baraquet C. Harwood C. S. Sondermann H. Navarro M. V. (2016). Mechanistic insights into c-di-GMP-dependent control of the biofilm regulator FleQ from *Pseudomonas aeruginosa*. Proc. Natl. Acad. Sci. U.S.A. 113, E209–E218. 10.1073/pnas.1523148113 26712005 PMC4720306

[B54] Mechold U. Fang G. Ngo S. Ogryzko V. Danchin A. (2007). YtqI from *Bacillus subtilis* has both oligoribonuclease and pAp-phosphatase activity. Nucl. Acids Res. 35, 4552–4561. 10.1093/nar/gkm462 17586819 PMC1935014

[B55] Mei H. Tang Q. Li X. Wang J. He J. (2015). Insights into sRNA genes regulated by two-component systems in the *Bacillus cereus* group. Curr. Bioinform. 10, 456–468. 10.2174/157489361004150922151028

[B56] Mendez-Ortiz M. M. Hyodo M. Hayakawa Y. Membrillo-Hernandez J. (2006). Genome-wide transcriptional profile of *Escherichia coli* in response to high levels of the second messenger 3',5'-cyclic diguanylic acid. J. Biol. Chem. 281, 8090–8099. 10.1074/jbc.M510701200 16418169

[B57] Mielich-Süss B. Lopez D. (2015). Molecular mechanisms involved in *Bacillus subtilis* biofilm formation. Environ. Microbiol. 17, 555–565. 10.1111/1462-2920.12527 24909922 PMC4188541

[B58] Mortazavi A. Williams B. A. McCue K. Schaeffer L. Wold B. (2008). Mapping and quantifying mammalian transcriptomes by RNA-Seq. Nat. Methods 5, 621–628. 10.1038/nmeth.1226 18516045 PMC13303166

[B59] Neusser T. Polen T. Geissen R. Wagner R. (2010). Depletion of the non-coding regulatory 6S RNA in *E. coli* causes a surprising reduction in the expression of the translation machinery. BMC Genomics 11:165. 10.1186/1471-2164-11-165 20222947 PMC2848244

[B60] Newell P. D. Boyd C. D. Sondermann H. O'Toole G. A. (2011). A c-di-GMP effector system controls cell adhesion by inside-out signaling and surface protein cleavage. PLoS Biol. 9:e1000587. 10.1371/journal.pbio.1000587. 21304920 PMC3032545

[B61] Newell P. D. Monds R. D. O'Toole G. A. (2009). LapD is a bis-(3',5')-cyclicdimeric GMP-binding protein that regulates surface attachment by *Pseudomonas fluorescens* Pf0-1. Proc. Natl. Acad. Sci. U.S.A. 106, 3461–3466. 10.1073/pnas.0808933106 19218451 PMC2651287

[B62] Palma L. Muñoz D. Berry C. Murillo J. Caballero P. (2014). *Bacillus thuringiensis* toxins: an overview of their biocidal activity. Toxins (Basel) 6, 3296–3325. 10.3390/toxins6123296 25514092 PMC4280536

[B63] Paul K. Nieto V. Carlquist W. Blair D. Harshey R. (2010). The c-di-GMP binding protein YcgR controls flagellar motor direction and speed to affect chemotaxis by a “backstop brake” mechanism. Mol. Cell 38, 128–167. 10.1016/j.molcel.2010.03.001 20346719 PMC2929022

[B64] Purcell E. B. McKee R. W. McBride S. M. Waters C. M. Tamayo R. (2012). Cyclic diguanylate inversely regulates motility and aggregation in *Clostridium difficile*. J. Bacteriol. 194, 3307–3316. 10.1128/JB.00100-12 22522894 PMC3434733

[B65] Qi M. Mei F. Wang H. Sun M. Wang G. Yu Z.. (2015). Function of global regulator CodY in *Bacillus thuringiensis* BMB171 by comparative proteomic analysis. J. Microbiol. Biotechnol. 25, 152–161. 10.4014/jmb.1406.06036 25223328

[B66] Rajeev L. Luning E. G. Altenburg S. Zane G. M. Baidoo E. E. Gatena M.. (2014). Identification of a cyclic-di-GMP-modulating response regulator that impacts biofilm formation in a model sulfate reducing bacterium. Front. Microbiol. 5:382. 10.3389/fmicb.2014.00382 25120537 PMC4114195

[B67] Rao F. See R. Y. Zhang D. Toh D. C. Ji Q. Liang Z. X. (2010). YybT is a signaling protein that contains a cyclic dinucleotide phosphodiesterase domain and a GGDEF domain with ATPase activity. J. Biol. Chem. 285, 473–482. 10.1074/jbc.M109.040238 19901023 PMC2804195

[B68] Raymond B. Bonsall M. B. (2013). Cooperation and the evolutionary ecology of bacterial virulence: the *Bacillus cereus* group as a novel study system. Bioessays 35, 706–716. 10.1002/bies.201300028 23702950

[B69] Raymond B. Johnston P. R. Nielsen-LeRoux C. Lereclus D. Crickmore N. (2010). *Bacillus thuringiensis*: an impotent pathogen? Trends Microbiol. 18, 189–194. 10.1016/j.tim.2010.02.006 20338765

[B70] Römling U. Galperin M. Y. Gomelsky M. (2013). Cyclic di-GMP: the first 25 years of a universal bacterial second messenger. Microbiol. Mol. Biol. Rev. 77, 1–52. 10.1128/MMBR.00043-12 23471616 PMC3591986

[B71] Ryan R. P. Fouhy Y. Lucey J. F. Crossman L. C. Spiro S. He Y. W.. (2006). Cell-cell signaling in *Xanthomonas campestris* involves an HD-GYP domain protein that functions in cyclic di-GMP turnover. Proc. Natl. Acad. Sci. U.S.A. 103, 6712–6717. 10.1073/pnas.0600345103 16611728 PMC1458946

[B72] Schmidt A. J. Ryjenkov D. A. Gomelsky M. (2005). The ubiquitous protein domain EAL is a cyclic diguanylate-specific phosphodiesterase: enzymatically active and inactive EAL domains. J. Bacteriol. 187, 4774–4781. 10.1128/JB.187.14.4774-4781.2005 15995192 PMC1169503

[B73] Schuhmacher J. S. Thormann K. M. Bange G. (2015). How bacteria maintain location and number of flagella? FEMS Microbiol. Rev. 39, 812–822. 10.1093/femsre/fuv034 26195616

[B74] Shanahan C. A. Gaffney B. L. Jones R. A. Strobel S. A. (2011). Differential analogue binding by two classes of c-di-GMP riboswitches. J. Am. Chem. Soc. 133, 15578–15592. 10.1021/ja204650q 21838307 PMC3183120

[B75] Simm R. Morr M. Kader A. Nimtz M. Römling U. (2004). GGDEF and EAL domains inversely regulate cyclic di-GMP levels and transition from sessility to motility. Mol. Microbiol. 53, 1123–1134. 10.1111/j.1365-2958.2004.04206.x 15306016

[B76] Slamti L. Lemy C. Henry C. Guillot A. Huillet E. Lereclus D. (2016). CodY regulates the activity of the virulence quorum sensor PlcR by controlling the import of the signaling peptide PapR in *Bacillus thuringiensis*. Front. Microbiol. 6:1501. 10.3389/fmicb.2015.01501 26779156 PMC4701985

[B77] Slamti L. Perchat S. Huillet E. Lereclus D. (2014). Quorum sensing in *Bacillus thuringiensis* is required for completion of a full infectious cycle in the insect. Toxins (Basel) 6, 2239–2255. 10.3390/toxins6082239 25089349 PMC4147580

[B78] Soberón M. López-Díaz J. A. Bravo A. (2013). Cyt toxins produced by *Bacillus thuringiensis*: a protein fold conserved in several pathogenic microorganisms. Peptides 41, 87–93. 10.1016/j.peptides.2012.05.023 22691603

[B79] Sondermann H. Shikuma N. J. Yildiz F. H. (2012). You've come a long way: c-di-GMP signaling. Curr. Opin. Microbiol. 15, 140–146. 10.1016/j.mib.2011.12.008 22226607 PMC3320698

[B80] Spangler C. Böhm A. Jenal U. Seifert R. Kaever V. (2010). A liquid chromatography-coupled tandem mass spectrometry method for quantitation of cyclic diguanosine monophosphate. J. Microbiol. Methods 81, 226–231. 10.1016/j.mimet.2010.03.020 20385176

[B81] Sudarsan N. Lee E. R. Weinberg Z. Moy R. H. Kim J. N. Link K. H.. (2008). Riboswitches in eubacteria sense the second messenger cyclic di-GMP. Science 321, 411–413. 10.1126/science.1159519 18635805 PMC5304454

[B82] Suppiger A. Eshwar A. K. Stephan R. Kaever V. Eberl L. Lehner A. (2016). The DSF type quorum sensing signalling system RpfF/R regulates diverse phenotypes in the opportunistic pathogen *Cronobacter*. Sci. Rep. 6:18753. 10.1038/srep18753 26725701 PMC4698668

[B83] Tang Q. Luo Y. Zheng C. Yin K. Ali M. K. Li X.. (2015). Functional Analysis of a c-di-AMP-specific Phosphodiesterase MsPDE from *Mycobacterium smegmatis*. Int. J. Biol. Sci. 11, 813–824. 10.7150/ijbs.11797 26078723 PMC4466462

[B84] Tang Q. Yin K. Qian H. Zhao Y. Wang W. Chou S. H.. (2016). Cyclic di-GMP contributes to adaption and virulence of *Bacillus thuringiensis* through a riboswitch-regulated collagen adhesion protein. Sci. Rep. 6:28807. 10.1038/srep28807 27381437 PMC4933901

[B85] Tao F. He Y. W. Wu D. H. Swarup S. Zhang L. H. (2010). The cyclic nucleotide monophosphate domain of *Xanthomonas campestris* global regulator Clp defines a new class of cyclic di-GMP effectors. J. Bacteriol. 192, 1020–1029. 10.1128/JB.01253-09 20008070 PMC2812978

[B86] Taylor B. L. Zhulin I. B. (1999). PAS domains: internal sensors of oxygen, redox potential, and light. Microbiol. Mol. Biol. Rev. 63, 479–506. 10357859 10.1128/mmbr.63.2.479-506.1999 PMC98974

[B87] Tuckerman J. R. Gonzalez G. Gilles-Gonzalez M. A. (2011). Cyclic di-GMP activation of polynucleotide phosphorylase signal-dependent RNA processing. J. Mol. Biol. 407, 633–639. 10.1016/j.jmb.2011.02.019 21320509

[B88] Ueda A. Wood T. K. (2009). Connecting quorum sensing, c-di-GMP, pel polysaccharide, and biofilm formation in *Pseudomonas aeruginosa* through tyrosine phosphatase TpbA (PA3885). PLoS Pathog. 5:e1000483. 10.1371/journal.ppat.1000483 19543378 PMC2691606

[B89] Wang F. He Q. Su K. X. Wei T. Xu S. Gu L. (2017). Structural and biochemical characterization of the catalytic domains of GdpP reveals a unified hydrolysis mechanism for the DHH/DHHA1 phosphodiesterase. Biochem. J. 475, 191–205. 10.1042/BCJ20170739 29203646

[B90] Wang H. Wang C. Yang K. Liu J. Zhang Y. Wang Y.. (2015). Genome wide distributions and functional characterization of copy number variations between Chinese and Western Pigs. PLoS ONE 10:e0131522. 10.1371/journal.pone.0131522 26154170 PMC4496047

[B91] Wang J. Mei H. Qian H. Tang Q. Liu X. Yu Z.. (2013a). Expression profile and regulation of spore and parasporal crystal formation-associated genes in *Bacillus thuringiensis*. J. Proteome Res. 12, 5487–5501. 10.1021/pr4003728 24215520

[B92] Wang J. Mei H. Zheng C. Qian H. Cui C. Fu Y. (2013b). The metabolic regulation of *Bacillus thuringiensis* for the formation of spores and parasporal crystals revealed by the transcriptomics and proteomics. Mol. Cell. Proteomics 12, 1363–1376. 10.1074/mcp.M112.023986 23408684 PMC3650345

[B93] Wang L. Feng Z. Wang X. Wang X. Zhang X. (2010). DEGseq: an R package for identifying differentially expressed genes from RNA-seq data. Bioinformatics 26, 136–138. 10.1093/bioinformatics/btp612 19855105

[B94] Wang Y. Chin K. Tu Z. He J. Jones C. Sanchez D.. (2016a). Nucleotide binding by the widespread high-affinity cyclic di-GMP receptor MshEN domain. Nat. Commun. 7:12481. 10.1038/ncomms12481 27578558 PMC5013675

[B95] Wang X. Li Z. Li X. Qian H. Cai X. Li X.. (2016b). Poly-β-hydroxybutyrate metabolism is unrelated to the sporulation and parasporal crystal protein formation in *Bacillus thuringiensis*. Front. Microbiol. 7:836. 10.3389/fmicb.2016.00836 27379025 PMC4908106

[B96] Weng J. Wang Y. Li J. Shen Q. Zhang R. (2013). Enhanced root colonization and biocontrol activity of *Bacillus amyloliquefaciens* SQR9 by *abrB* gene disruption. Appl. Microbiol. Biotechnol. 97, 8823–8830. 10.1007/s00253-012-4572-4 23196984

[B97] Whiteley A. T. Garelis N. E. Peterson B. N. Choi P. H. Tong L. Woodward J. J.. (2017). c-di-AMP modulates *Listeria monocytogenes* central metabolism to regulate growth, antibiotic resistance and osmoregulation. Mol. Microbiol. 104, 212–233. 10.1111/mmi.13622 28097715 PMC5391996

[B98] Woodward J. J. Iavarone A. T. Portnoy D. A. (2010). c-di-AMP secreted by intracellular *Listeria monocytogenes* activates a host type I interferon response. Science 328, 1703–1705. 10.1126/science.1189801 20508090 PMC3156580

[B99] Xin B. Zheng J. Xu Z. Song X. Ruan L. Peng D.. (2015). The *Bacillus cereus* group is an excellent reservoir of novel lanthipeptides. Appl. Environ. Microbiol. 81, 1765–1774. 10.1128/AEM.03758-14 25548056 PMC4325169

[B100] Xu C. Wang B. C. Yu Z. Sun M. (2014). Structural insights into *Bacillus thuringiensis* Cry, Cyt and parasporin toxins. Toxins (Basel). 6, 2732–2770. 10.3390/toxins6092732 25229189 PMC4179158

[B101] Yang F. Tian F. Chen H. Hutchins W. Yang C. H. He C. (2015). The Xanthomonas oryzae pv. *oryzae* PilZ domain proteins function differentially in cyclic di-GMP binding and regulation of virulence and motility. Appl. Environ. Microbiol. 81, 4358–4367. 10.1128/AEM.04044-14 25911481 PMC4475898

[B102] Yang H. He J. Hu F. Zheng C. Yu Z. (2010). Detection of *Escherichia coli* enoyl ACP reductase using biarsenical-tetracysteine motif. Bioconjug. Chem. 21, 1341–1348. 10.1021/bc1001533 20583783

[B103] Ye J. Coulouris G. Zaretskaya I. Cutcutache I. Rozen S. Madden T. L. (2012). Primer-BLAST: a tool to design target-specific primers for polymerase chain reaction. BMC Bioinformatics 13:134. 10.1186/1471-2105-13-134 22708584 PMC3412702

[B104] Zheng C. Ma Y. Wang X. Xie Y. Ali M. K. He J. (2015). Functional analysis of the sporulation-specific diadenylate cyclase CdaS in *Bacillus thuringiensis*. Front. Microbiol. 6:908. 10.3389/fmicb.2015.00908 26441857 PMC4568413

[B105] Zheng C. Wang J. Luo Y. Fu Y. Su J. He J. (2013). Highly efficient enzymatic preparation of c-di-AMP using the diadenylate cyclase DisA from *Bacillus thuringiensis*. Enzyme Microb. Technol. 52, 319–324. 10.1016/j.enzmictec.2013.03.007 23608499

[B106] Zhou H. Zheng C. Su J. Chen B. Fu Y. Xie Y.. (2016). Characterization of a natural triple-tandem c-di-GMP riboswitch and application of the riboswitch-based dual-fluorescence reporter. Sci. Rep. 6:20871. 10.1038/srep20871 26892868 PMC4759541

[B107] Zhu Y. Yuan Z. Gu L. (2017). Structural basis for the regulation of chemotaxis by MapZ in the presence of c-di-GMP. Acta Crystallogr. D. Struct. Biol. 73, 683–691. 10.1107/S2059798317009998 28777083

[B108] Zhulin I. B. Taylor B. L. Dixon R. (1997). PAS domain S-boxes in archaea, bacteria and sensors for oxygen and redox. Trends Biochem. Sci. 22, 331–333. 9301332 10.1016/s0968-0004(97)01110-9

