# Supplementary material for: c-di-GMP Regulates Various Phenotypes and Insecticidal Activity of Gram-Positive Bacillus thuringiensis
Source: Front Microbiol. 2018 Feb 13;9:45. doi: 10.3389/fmicb.2018.00045 (PMC5816809; doi:10.3389/fmicb.2018.00045)
Supplement: Supplementary file 1 [file Table1.doc]

**Table S1.** **The list of strains and plasmids used in this study**

| **Strain and plasmid** | **Description** | **Reference** |
| --- | --- | --- |
| **Strains** |  |  |
| *E. coli*BL21(DE3) | Protein expression host | Novagen |
| BL21/pET28b-*RS02850* | BL21(DE3) with pET28b-*RS02850*, used for purifying protein RS02850 | This work |
| BL21/pET28b-*RS03240* | BL21(DE3) with pET28b-*RS03240*, used for purifying protein RS03240 | This work |
| BL21/pET28b-*RS17435_GGDEF-EAL_* | BL21(DE3) with pET28b-*RS17435_GGDEF-EAL_*, used for purifying protein RS17435 which was purified with their transmembrane domains truncated. | This work |
| BL21/pET28b-*RS18570_GGDEF-EAL_* | BL21(DE3) with pET28b-*RS18570_GGDEF-EAL_*, used for purifying protein RS18570 which was purified with their transmembrane domains truncated. | This work |
| BL21/pET28b-*RS19795* | BL21(DE3) with pET28b-*RS19795*, used for purifying protein RS19795 | This work |
| BL21/pET28b-*RS19835_GGDEF_* | BL21(DE3) with pET28b-*RS19835_GGDEF_*, used for purifying protein RS19835 which was purified with their transmembrane domains truncated. | This work |
| BL21/pET28b-*RS20080_GGDEF_* | BL21(DE3) with pET28b-*RS20080_GGDEF_*, used for purifying protein RS20080 which was purified with their transmembrane domains truncated. | This work |
| BL21/pET28b-*RS26115_GGDEF_* | BL21(DE3) with pET28b-*RS26115_GGDEF_*, used for purifying protein RS26115 which was purified with their transmembrane domains truncated. | This work |
| BL21/pET28b-*RS26475_GGDEF-EAL_* | BL21(DE3) with pET28b-*RS26475_GGDEF-EAL_*, used for purifying protein RS26475 which was purified with their transmembrane domains truncated. | This work |
| BL21/pET28b-*RS26720_GGDEF-EAL_* | BL21(DE3) with pET28b-*RS26720_GGDEF-EAL_*, used for purifying protein RS26720 which was purified with their transmembrane domains truncated. | This work |
| BL21/pET28b-*RS27040_GGDEF_* | BL21(DE3) with pET28b-*RS27040_GGDEF_*, used for purifying protein RS27040 which was purified with their transmembrane domains truncated. | This work |
| BL21/pET28b-*RS28330_GGDEF-EAL_* | BL21(DE3) with pET28b-*RS28330_GGDEF-EAL_*, used for purifying protein RS28330 which was purified with their transmembrane domains truncated. | This work |
| BL21/pET28b-*pleD* | BL21(DE3) with pET28b-*pleD*,used for purifying protein PleD | Zhou *et al*.,2016 |
| BL21/pET28b-*pleD*& pRP0122-Pbe-*amcyan*_*Bc3-5*_*turborfp* | BL21(DE3) with pET28b-*pleD* and pRP0122-Pbe-*amcyan*_*Bc3-5*_*turborfp*, used for DGC activity of PleD as CK^+^by dual-fluorescence reporter | Zhou *et al*.,2016 |
| BL21/pET28b& pRP0122-Pbe-*amcyan*_*Bc3-5*_*turborfp* | BL21(DE3) with pET28b and pRP0122-Pbe-*amcyan*_*Bc3-5*_*turborfp*, used for DGC activity of PleD as CK^-^ by dual-fluorescence reporter | Zhou *et al*.,2016 |
| BL21/pET28b-*RS19835_GGDEF_*& pRP0122-Pbe-*amcyan*_*Bc3-5*_*turborfp* | BL21(DE3) with pET28b-*RS19835_GGDEF_* and pRP0122-Pbe-*amcyan*_*Bc3-5*_*turborfp*, used for DGC activity of *RS19835* by dual-fluorescence reporter | This work |
| BL21/pET28b-*RS20080_GGDEF_*& pRP0122-Pbe-*amcyan*_*Bc3-5*_*turborfp* | BL21(DE3) with pET28b-*RS20080_GGDEF_* and pRP0122-Pbe-*amcyan*_*Bc3-5*_*turborfp*, used for DGC activity of *RS20080* by dual-fluorescence reporter | This work |
| BL21/pET28b-*RS26115_GGDEF_*& pRP0122-Pbe-*amcyan*_*Bc3-5*_*turborfp* | BL21(DE3) with pET28b-*RS26115_GGDEF_* and pRP0122-Pbe-*amcyan*_*Bc3-5*_*turborfp*, used for DGC activity of *RS20080* by dual-fluorescence reporter | This work |
| BL21/pET28b-*RS27040_GGDEF_*& pRP0122-Pbe-*amcyan*_*Bc3-5*_*turborfp* | BL21(DE3) with pET28b-*RS27040_GGDEF_* and pRP0122-Pbe-*amcyan*_*Bc3-5*_*turborfp*, used for DGC activity of *RS27040* by dual-fluorescence reporter | This work |
| BL21/pET28b-*RS02850*& pRP0122-Pbe-*amcyan*_*Bc3-5*_*turborfp* | BL21(DE3) with pET28b-*RS02850* and pRP0122-Pbe-*amcyan*_*Bc3-5*_*turborfp*, used for DGC activity of *RS02850* by dual-fluorescence reporter | This work |
| BL21/pET28b-*RS03240*& pRP0122-Pbe-*amcyan*_*Bc3-5*_*turborfp* | BL21(DE3) with pET28b-*RS03240* and pRP0122-Pbe-*amcyan*_*Bc3-5*_*turborfp*, used for DGC activity of *RS03240* by dual-fluorescence reporter | This work |
| BL21/pET28b-*RS17435_GGDEF+EAL_*& pRP0122-Pbe-*amcyan*_*Bc3-5*_*turborfp* | BL21(DE3) with pET28b-*RS17435_GGDEF+EAL_* and pRP0122-Pbe-*amcyan*_*Bc3-5*_*turborfp*, used for DGC activity of *RS17435* by dual-fluorescence reporter | This work |
| BL21/pET28b-*RS18570_GGDEF+EAL_*& pRP0122-Pbe-*amcyan*_*Bc3-5*_*turborfp* | BL21(DE3) with pET28b-*RS18570_GGDEF+EAL_* and pRP0122-Pbe-*amcyan*_*Bc3-5*_*turborfp*, used for DGC activity of *RS18570* by dual-fluorescence reporter | This work |
| BL21/pET28b-*RS26720_GGDEF+EAL_*& pRP0122-Pbe-*amcyan*_*Bc3-5*_*turborfp* | BL21(DE3) with pET28b-*RS26720_GGDEF+EAL_* and pRP0122-Pbe-*amcyan*_*Bc3-5*_*turborfp*, used for DGC activity of *RS26720* by dual-fluorescence reporter | This work |
| BL21/pET28b-*RS26475_GGDEF+EAL_*& pRP0122-Pbe-*amcyan*_*Bc3-5*_*turborfp* | BL21(DE3) with pET28b-*RS26475_GGDEF+EAL_* and pRP0122-Pbe-*amcyan*_*Bc3-5*_*turborfp*, used for DGC activity of *RS26475* by dual-fluorescence reporter | This work |
| BL21/pET28b-*RS28330_GGDEF+EAL_*& pRP0122-Pbe-*amcyan*_*Bc3-5*_*turborfp* | BL21(DE3) with pET28b-*RS28330_GGDEF+EAL_* and pRP0122-Pbe-*amcyan*_*Bc3-5*_*turborfp*, used for DGC activity of *RS28330* by dual-fluorescence reporter | This work |
| *E. coli*DH5α | A cloning host | Novagen |
| DH5α/pSS1827 | DH5α with pSS1827, used for knock out of gene in *B.thuringiensis* | Janes and Stibitz, 2006 |
| DH5α/pSS4332 | DH5α with pSS4332, used for knock out of gene in *B.thuringiensis* | Janes and Stibitz, 2006 |
| DH5α/pRP1028 | DH5α with pRP1028 used for knock out of gene in *B.thuringiensis* | Janes and Stibitz, 2006 |
| DH5α/pRP1028-*RS03240*UD | DH5α with pRP1028-*RS03240*UD, used for knock out of *RS03240* in *B.thuringiensis* | This work |
| DH5α/pRP1028-*RS02850*UD | DH5α with pRP1028-*RS02850*UD, used for knock out of *RS02850* in *B.thuringiensis* | This work |
| DH5α/pRP1028-*RS18570*UD | DH5α with pRP1028-*RS18570*UD, used for knock out of *RS18570* in *B.thuringiensis* | This work |
| *B. thuringiensis*BMB171 | An acrystalliferous mutant strain with high transformation frequency (NC_014171) | Stored by our lab |
| Δ1*pde* | *RS03240*mutant of BMB171 | This work |
| Δ2*pde* | *RS02850*and*RS03240*double mutant of BMB171 | This work |
| Δ3*pde* | *RS02850*,*RS03240*and*RS18570*triple mutant of BMB171 | This work |
| BMB171-*cry1Ac* | BMB171 containing plasmid pBMB43-304, which expressed Cry1Ac10 in BMB171 | This work |
| Δ3*pde*-*cry1Ac* | Δ3*pde* containing plasmid pBMB43-304, which expressed Cry1Ac10 in Δ3*pde* | This work |
| **Plasmids** |  |  |
| pET28b(+) | T7 promoter expression vector, *kan^r^* | Novagen |
| pET28b-*RS02850* | *RS02850* in *Nco*I and *Xho*I sites of pET28b,used for purifying protein RS02850 | This work |
| pET28b-*RS03240* | *RS03240*in *Nco*I and *Xho*I sites of pET28b,used for purifying protein RS03240 | This work |
| pET28b-*RS17435_GGDEF-EAL_* | *RS17435_GGDEF-EAL_*in *Nco*I and *Xho*I sites of pET28b, used for purifying protein RS17435 which was purified with their transmembrane domains truncated. | This work |
| pET28b-*RS18570_GGDEF-EAL_* | *RS18570_GGDEF-EAL_*in *Nco*I and *Xho*I sites of pET28b, used for purifying protein RS18570 which was purified with their transmembrane domains truncated. | This work |
| pET28b-*RS19795* | *RS19795*in *Nco*I and *Xho*I sites of pET28b,used for purifying protein RS19795 | This work |
| pET28b-*RS19835_GGDEF_* | *RS19835_GGDEF_*in *Nco*I and *Xho*I sites of pET28b,used for purifying protein RS19835 which was purified with their transmembrane domains truncated. | This work |
| pET28b-*RS20080_GGDEF_* | *RS20080_GGDEF_*in *Nco*I and *Xho*I sites of pET28b,used for purifying protein RS20080 which was purified with their transmembrane domains truncated. | This work |
| pET28b-*RS26115_GGDEF_* | *RS26115_GGDEF_*in *Nco*I and *Xho*I sites of pET28b,used for purifying protein RS26115 which was purified with their transmembrane domains truncated. | This work |
| pET28b-*RS26475_GGDEF-EAL_* | *RS26475_GGDEF-EAL_*in *Nco*I and *Xho*I sites of pET28b, used for purifying protein RS26475 which was purified with their transmembrane domains truncated. | This work |
| pET28b-*RS26720_GGDEF-EAL_* | *RS26720_GGDEF-EAL_*in *Nco*I and *Hind*III sites of pET28b, used for purifying protein RS26720 which was purified with their transmembrane domains truncated. | This work |
| pET28b-*RS27040_GGDEF_* | *RS27040_GGDEF_*in *Nco*I and *Xho*I sites of pET28b,used for purifying protein RS27040 which was purified with their transmembrane domains truncated. | This work |
| pET28b-*RS28330_GGDEF-EAL_* | *RS28330_GGDEF-EAL_*in *Nco*I and *Xho*I sites of pET28b, used for purifying protein RS28330 which was purified with their transmembrane domains truncated. | This work |
| pET28b-*pleD* | *pleD* in *Nco*I and *Xho*I sites of pET28b,used for purifying protein PleD | Zhou *et al*.,2016 |
| pET11a-*pleD* | pET11a carrying *pleD*, used for expression vector construction | Chan *et al.*, 2004 |
| pSS1827 | the helper plasmid for transconjugation, *amp^r^* | Janes and Stibitz, 2006 |
| pSS4332 | *B. thuringiensis*-*E. coli* shuttle plasmid; containing *gfp* I-SceI restriction enzyme encoding genes, *kan^r^* | Janes and Stibitz, 2006 |
| pRP1028 | *B. thuringiensis*-*E. coli* shuttle plasmid; containing *turbo-rfp* gene and an I-SceI recognition site, *amp^r^spc^r^* | Janes and Stibitz, 2006 |
| pRP1028-*RS03240*UD | pRP1028 with the upstream and downstream regions of *RS03240*, used for *RS03240* deletion | This work |
| pRP1028-*RS02850*UD | pRP1028 with the upstream and downstream regions of *RS02850*, used for *RS02850* deletion | This work |
| pRP1028-*RS18570*UD | pRP1028 with the upstream and downstream regions of *RS18570*, used for *RS18570* deletion | This work |
| pBMB43-304 | pHT304 carrying *cry1Ac10*, used for expression of Cry1Ac10 protein in *B. thuringiensis* | Qi *et al.*, 2015 |
| pRP0122-Pbe-*amcyan*_*Bc3-5*_*turborfp* | The plasmid for biosenser | Zhou *et al*.,2016 |
