# Supplementary material for: c-di-GMP Regulates Various Phenotypes and Insecticidal Activity of Gram-Positive Bacillus thuringiensis
Source: Front Microbiol. 2018 Feb 13;9:45. doi: 10.3389/fmicb.2018.00045 (PMC5816809; doi:10.3389/fmicb.2018.00045)
Supplement: Supplementary file 2 [file Table2.doc]

**Table S2. Sequences of oligonucleotide primers used in this study**

| **Name** | **Sequence (5’ to 3’)** |
| --- | --- |
| *Cloning primers for expression* | |
| *RS02850*F  *RS02850*R | CTGACCATGGGGAAGGAAGAATATAGTAATC CTGACTCGAGTTTATAATACATATTTTCCATATC |
| *RS03240*F  *RS03240*R | CTGACCATGGGGCTAGAACAGAGAGGTCATGC CTGACTCGAGAAAATCTGTAATTAACTTTTCTTTTAGC |
| *RS17435*F  *RS17435*R | CTGACCATGGAATTAGATAGATCCATTAAGCG CTGACTCGAGTTCATTTTGCATTGTACTTAAAAAATCG |
| *RS18570*F  *RS18570*R | CTGACCATGGTGAAAGATATTACTGC CTGACTCGAGTCGGTGAATGCCGTTTTCTAATAG |
| *RS19795*F  *RS19795*R | CTGACCATGGGGTTGATAGATGCATTAGATGTAATGG CTGACTCGAGAAATAAAACATCTTCTTCATATAAATT |
| *RS19835*F  *RS19835*R | CTGACCATGGGGTATTATTTTGGACATTGGTACG CTGACTCGAGCTCATTTTCTTGCAAATTTCTATAC |
| *RS20080*F  *RS20080*R | CTGACCATGGGGTTTATAGCAAATAGTTTTGAAGAG CTGACTCGAGCATTTTTTCTTTTCTATACACAC |
| *RS26115*F  *RS26115*R | CTGACCATGGGGACGACTGCAATGAATCGTCG  CTGACTCGAGTAACAGTGTCTGTCCTTTGTTTC |
| *RS26475*F  *RS26475*R | CTGACCATGGGGATTAAAGATATTACGGAAAGC CTGACTCGAGTTTGTTCATACTGGGGGTTTTTTG |
| *RS26720*F  *RS26720*R | CTGACCATGGATGCACTTACTGGATTACC CTGAAGCTTGACGGTTGTTTTTTTATGGAGTAAC |
| *RS27040*F  *RS27040*R | CTGACCATGGAGCTATTTCAAACGATGAAGC CTGACTCGAGAGATGCAGAGCTGACCTTATTTC |
| *RS28330*F  *RS28330*R | CTGACCATGGGGAAACAACAACAGGAAGAGATA  CTGACTCGAGTTATTCATTTTGCATTACAATTAAA |
| *Cloning primers for mutant construction* | |
| *URS02850*F  *URS02850*R | CGAAGCTTTACATTTGATTAGACCGCTTGGA CGACGCGTATTTATCTCAACCCGTCTTATCT |
| *DRS02850*F  *DRS02850*R | CGACGCGTCAAGGTTATTATTATAGCCGACC CGGGATCCATTGGGCATCCTTTAATTGC |
| *URS03240*F  *URS03240*R | CGACGCGTTAAAAAAGAGAAGCCAGCAGC CGGGATCCCTCTAATTTCGGAGACGGTC |
| *DRS03240*F  *DRS03240*R | CGACGCGTTAAAAAAGAGAAGCCAGCAGC CGGGATCCCTCTAATTTCGGAGACGGTC |
| *URS18570*F  *URS18570*R | CGAAGCTTTCTTTCTGTGGAAGTGAGCGT CGACGCGTGAAGCGTACATCTGAATTTGA |
| *DRS18570*F  *DRS18570*R | CGACGCGTCTATTAGAAAACGGCATTCACC CGGGATCCGCTATAGGAGCATGGTTAAATCG |
| *URS19795*F  *URS19795*R | CGAAGCTTATAACAGGTGGATCGAGTGG CGACGCGTCGTGCTTCCCTCCCTTTACTTTTC |
| *DRS19795*F  *DRS19795*R | CGACGCGTTGCACGAAGAGATTCGTG CGGGATCCCCTGACTTTCATGATATCCTGC |
| *Detection primers for mutant construction* | |
| *MRS03240*F  *MRS03240*R | CAGATAGAAGCAAGGAGAGATGCGC GCGTATAGTTTCGCTATCGTCGTA |
| *MRS18570*F  *MRS18570*R | TCAAATTCAGATGTACGCTTC GCTATAGGAGCATGGTTAAATCG |
| *MRS19795*F  *MRS19795*R | CAGGTCCAGGAGTTATTCATTCG GCAACACATACAAATAGAACAAGCC |
| *UURS02850*F | TTTTATATCAGCCAGAAATTCCAGC |
| *UURS03240*F | ATCATGGAGGACACAATCATGG |
| *UURS18570*F | CATCTAACCCAGGCTTAATTTC |
| *UURS19795*F | TTTGGAGGGGATGTATGTGAAAG |
| *1028I*R | GTGCGAATAAGGGACAGTGAAGAAGAAGG |
| *Reverse Transcription PCR Primers* | |
| *RRS19835*F  *RRS19835*R | ACGAGCGTATGGGTATGGAAG  CATCGCCCTTCCGAATTGTC |
| *RRS20080*F  *RRS20080*R | ATAGCCCCTGTAACTCCGCA  AAATGTTGCTCGCGATAGGC |
| *RRS26115*F  *RRS26115*R | CGACTGCAATGAATCGTCGT  TACGATGTCAGCACCAGCTT |
| *RRS27040*F  *RRS27040*R | GTGGAGAGGAGTTTGCGCTT  GTATAACGCATCATCCGCCG |
| *RRS19795*F  *RRS19795*R | TATGCTTGGAAGAACGGCGG  CATTATATTCTCGAGAAAATAC |
| *RRS02850*F  *RRS02850*R | GCAAATCCGGCAACGTATGA  GAAATATATAGCTCGTACCC |
| *RRS03240*F  *RRS03240*R | ACCATTTATGCTGGAAGGCCA  CGCGCGTAGCTCATCTGTA |
| *RRS17435*F  *RRS17435*R | TTCCATGATACGGTGACGGA  CTTGCTAGAGGAATAGT |
| *RRS18570*F  *RRS18570*R | ACGAAAATTGGTAACCGTGCC  GATTGTAAATTCATCGCCAC |
| *RRS26475*F  *RRS26475*R | TGCGCTATCCATTTTGCAGC  GTATTAAAGCTTCAGCACCG |
| *RRS26720*F  *RRS26720*R | CGGAATTGCGATGTACCCTG  CTTGCGGCTGGTATTCAAGG |
| *RRS28330*F  *RRS28330*R | AGGGTGATTTGTCAGAGCGT  GCTGAAGAAGTTTGCTCTTTG |
| *Real Time PCR* *Primers* | |
| *QCRS00200*F  *QCRS00200*R | TTAGGTCGTGTAGTAATTCCAATCG  TAAGATTTGCTCAGCGCCTTC |
| *QCRS02135F*  *QCRS02135R* | CTGTTGTGAGTGCGATTGCTAG  TGTTGTACTTCATCTTGCATATTCC |
| *QCRS02220F*  *QCRS02220R* | CTGTTGTGAGTGCGATTGCTAG  AGAGACATGGCGAAAGATCCTAC |
| *QCRS02905F*  *QCRS02905R* | ATCGAAGAAGATTAGCGAAGGTG  GCTTCGCTTGTCTGCTTCATAC |
| *QCRS06745*F  *QCRS06745*R | TATTGCTTGTTATCCTACCGACAAT  TTACCAGTCTCGGTGCCTACAAC |
| *QCRS06770*F  *QCRS06770*R | GCCTATTGCTGGTGGTTCTATCTTA  TTGAAAGGAAGAATGGTATCCAACT |
| *QCRS08250F*  *QCRS08250R* | AGTAGACGATGCGATGTTTATGCG  ATTTCTTTTAATGCTTCAAGCCCG |
| *QCRS08255F*  *QCRS08255R* | TTCCGCTCCGCTCATACATTC  TTGCCCATTCCACCTTGTTGC |
| *QCRS08375F*  *QCRS08375R* | AAAAGGCTTCGAAAACTACACTAAT  ATACGGTTCTTCACCAGCAGC |
| *QCRS08395F*  *QCRS08395R* | TTACGAATCTGCTGGAGGGAG  TTTTTACCTGTCGTTCCTACCATAG |
| *QCRS08400*F  *QCRS08400*R | AACATTCATTGGCAAATATGGAGC  ATCAATCTCTACACCAATAGCCTGC |
| *QCRS08405*F  *QCRS08405*R | GCTCCACTTACTCCTTCAAAAACTC  AGTCGATGTGAAAAATAGTTGATCC |
| *QCRS08435F*  *QCRS08435R* | AAAAGAAATGAATGGGATGGGC  CAATGTAATTTGAACATTTGCCG |
| *QCRS08450F*  *QCRS08450R* | TTTTGCTGGGTCAGGTGTTGG  GATGACTTTCATCTGACGTCGCTAC |
| *QCRS08470*F  *QCRS08470*R | GAGACAGCAATAGAGGGAAATGG  ATTGGAATATGAATTGGACTTGGTC |
| *QCRS08485F*  *QCRS08485R* | CCTGTTACAACTGTGCCAGAATC  CCACGAAATACGTTGAATACGATG |
| *QCRS08505*F  *QCRS08505*R | TACTGGTGACGCTGCTTTGGG  GGGAGGAGTGGCAGTTGTATCTAAG |
| *QCRS08525F*  *QCRS08525R* | AATGTTGGCTTGGCGGAGATAG  TGATATCGCTCGCTGTCGTAATC |
| *QCRS08530F*  *QCRS08530R* | TGAAGCATGAAGTATCTCCTGTGTC  TCTACTTCAAGAACATCGCCAAC |
| *QCRS09855*F  *QCRS09855*R | AATTGTTATCCCTAAAGAATTACGC  CGCTCCCTCTATCCCAACAGT |
| *QCRS13095*F  *QCRS13095*R | CCCTTTGTTCTAGTATTCCGCTTAT  GCTGTAACGATGAACATTATTCCGT |
| *QCRS15405*F  *QCRS15405*R | TCGAAAAGCTCTATGGAGGGAT  GAGGTTCCCAATACTCTCCAATG |
| *QCRS19885*F  *QCRS19885*R | GAAATGCCTCCGAAGCCAAAT  AAAACTGGAAGAAAGCTCATTCCCT |
| *QCRS20745*F  *QCRS20745*R | CATCATTCCGATCAGCAACAAC  GCGATATCTGGATACAATACTTTCG |
| *QCRS22145*F  *QCRS22145*R | TTCAAGACATGATTGGCTCAATC  TGTAATTTCCCTAATACTTCGTACTCC |
| *QCRS22440F*  *QCRS22440R* | TTACTTGTTTCAGCGTCACCTCG  GTATTCTCCGTATCCAATCGCAC |
| *QCRS22445F*  *QCRS22445R* | TATGAAATTTGTGATTGATGGCG  TAGCATGACCTAACTTTTCAATATCAG |
| *QCRS24510*F  *QCRS24510*R | AAATTGGCGAAATCGTTACTGGT  ATGTTCGTCTGTTCACGGAAAGC |
| *QCRS25150F*  *QCRS25150R* | GACGGAGATTTAACGGCTAGATTAC  AGAGCATTCATAGTTTCTTGGGTTC |
| *QCRS26660*F  *QCRS26660*R | GAAGGTTATCAGACGTTCCAAGC  GGATCATATCAAGCTCTCCATAAGC |

Underlined: restriction sites F: Forward primers R: Reverse primers

U: upstream/upstream arms D: downstream/downstream arms

M: primer location in the middle of genes Q: RT-QPCR
