# Supplementary material for: c-di-GMP Regulates Various Phenotypes and Insecticidal Activity of Gram-Positive Bacillus thuringiensis
Source: Front Microbiol. 2018 Feb 13;9:45. doi: 10.3389/fmicb.2018.00045 (PMC5816809; doi:10.3389/fmicb.2018.00045)
Supplement: Supplementary file 6 [file Image1.pdf]

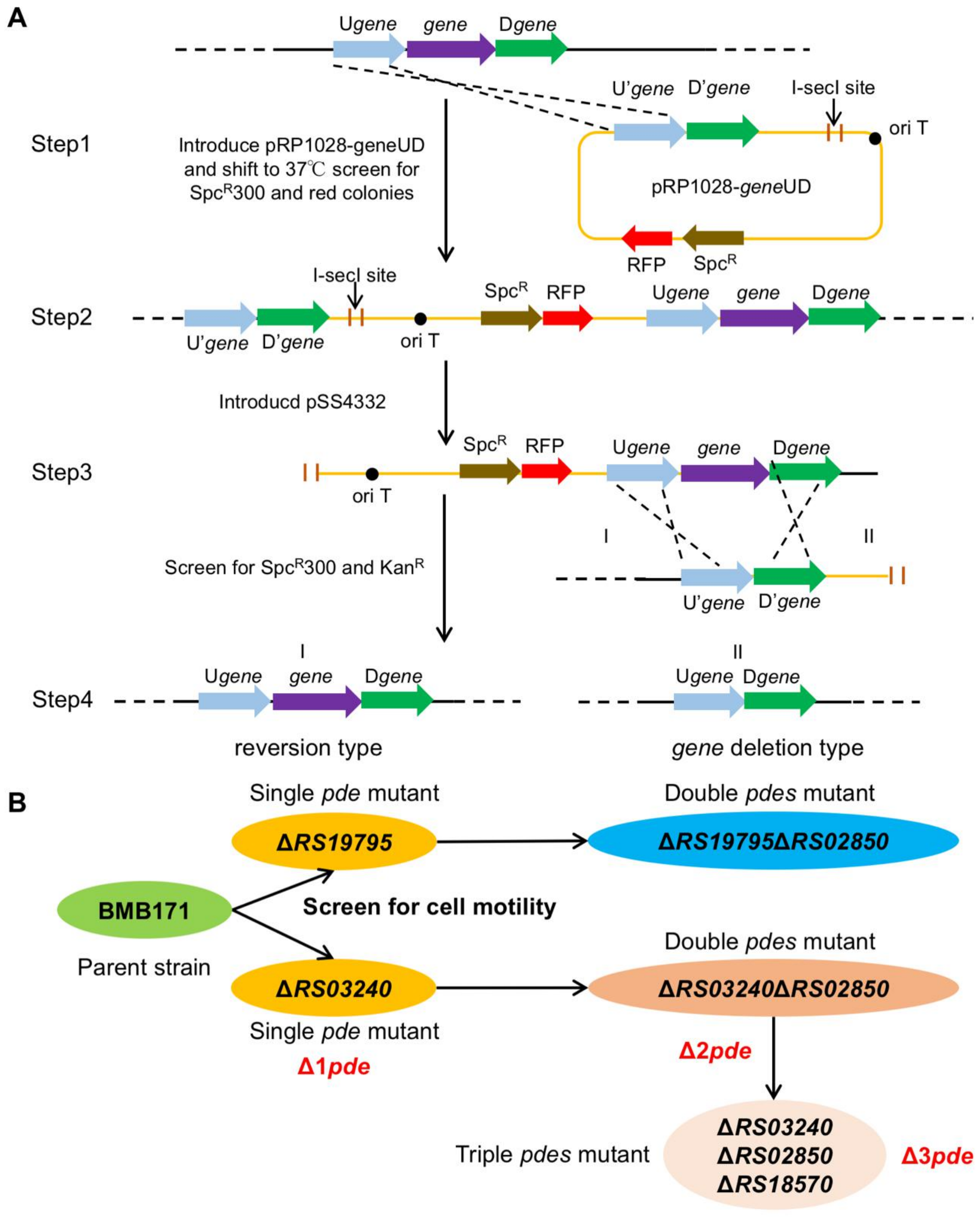

Figure S1. (A) Schemes of markerless gene knock-out procedures in *B. thuringiensis* BMB171. This figure is taken from the previous study in our laboratory (Zheng et al., 2015). **(B) Screening process of several *pde* genes deletion mutants.** Four candidate genes (*RS02850*, *RS03240*, *RS18570* and *RS19795*) with high transcription and high PDE activity *in vitro* were selected to construct different mutants in our study. Using cell motility as the screening criteria, two single *pde* gene deletion mutants Δ*RS19795* and

Δ*RS03240* were constructed in the first step, and then *RS02850* was selected to construct the two double *pde* genes deletion mutants Δ*RS19795*Δ*RS02850* and Δ*RS03240*Δ*RS02850*. Subsequently, *RS18570* was deleted from Δ*RS03240*Δ*RS02850* to obtain a relative high c-di-GMP concentration mutant Δ*RS03240*Δ*RS02850*Δ*RS18570*. The names of Δ1*pde*, Δ2*pde* and Δ3*pde* will be used to refer to the single *pde* gene deletion mutant Δ*RS03240*, the double *pde* genes deletion mutant Δ*RS03240*Δ*RS02850* and the triple *pde* genes deletion mutant Δ*RS03240*Δ*RS02850*Δ*RS18570*, respectively.
