# Supplementary material for: c-di-GMP Regulates Various Phenotypes and Insecticidal Activity of Gram-Positive Bacillus thuringiensis
Source: Front Microbiol. 2018 Feb 13;9:45. doi: 10.3389/fmicb.2018.00045 (PMC5816809; doi:10.3389/fmicb.2018.00045)
Supplement: Supplementary file 7 [file Image2.pdf]

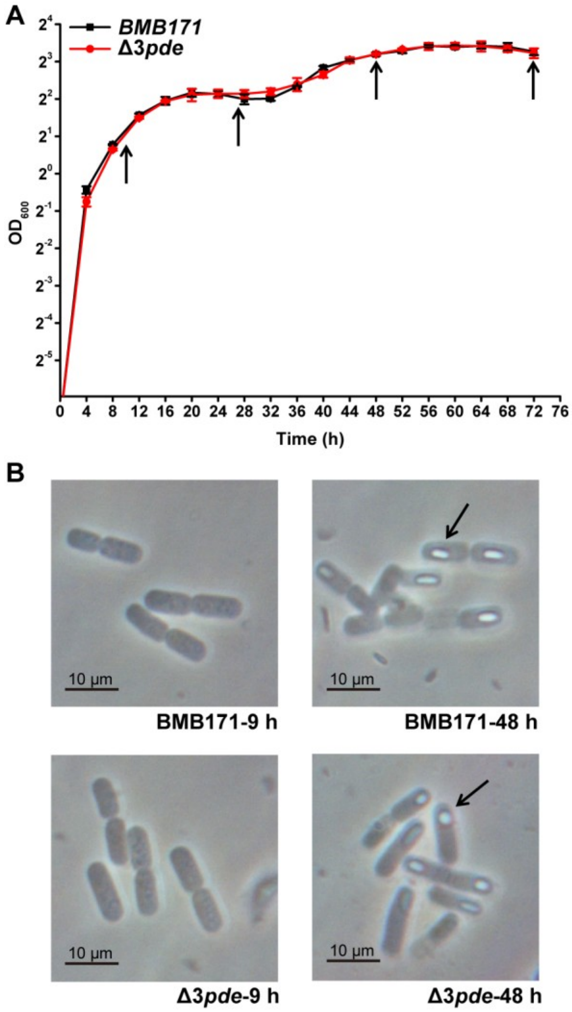

Figure S2. Growth features of BMB171 and Δ3*pde* in LB medium. (A) The growth curves of strains BMB171 and Δ3*pde* in LB medium. The Y-axis represents the average optical densities of triplicate bacterial cultures at 600 nm at each time point. Data are the averages of three independent experiments (error bars are standard error of mean values). The black arrows indicate the four sampling time points at 9 h, 27 h, 48 h and 72 h. BMB171 and Δ3*pde* samples were applied not only for detecting the intracellular concentration of c-di-GMP and for the virulence assay against cotton bollworm larvae at 9 h and 48 h, but also for RNA-seq analysis at 9 h. **(B)** Images of cell growth status at two time points of 9 h and 48 h. Pictures of the two sampling points at 9 h and 48 h were taken using a Nikon ECLIPSE E6000 phase contrast microscope (Nikon, Japan). The spores were marked by the black arrows. Scale bars represent 10 micrometers. The growth features of Δ3*pde* did not exhibit any visible difference compared with that of BMB171. The exponential phase is from 0 h to 16 h, the stationary phase from 16 h to 36 h, and the sporulation phase from 56 h to 72 h. The transitional period from 36 h to 56 h is of particular interest compared with other growth phases. *B. thuringiensis* exhibits a

secondary growth phase possibly due to the “cannibalism” and metabolism of some amino acids (Wang *et al.*, 2013a,b). To be exact, the so-called secondary growth phase is the late stationary phase or early sporulation phase.
