# Supplementary material for: c-di-GMP Regulates Various Phenotypes and Insecticidal Activity of Gram-Positive Bacillus thuringiensis
Source: Front Microbiol. 2018 Feb 13;9:45. doi: 10.3389/fmicb.2018.00045 (PMC5816809; doi:10.3389/fmicb.2018.00045)
Supplement: Supplementary file 8 [file Image3.pdf]

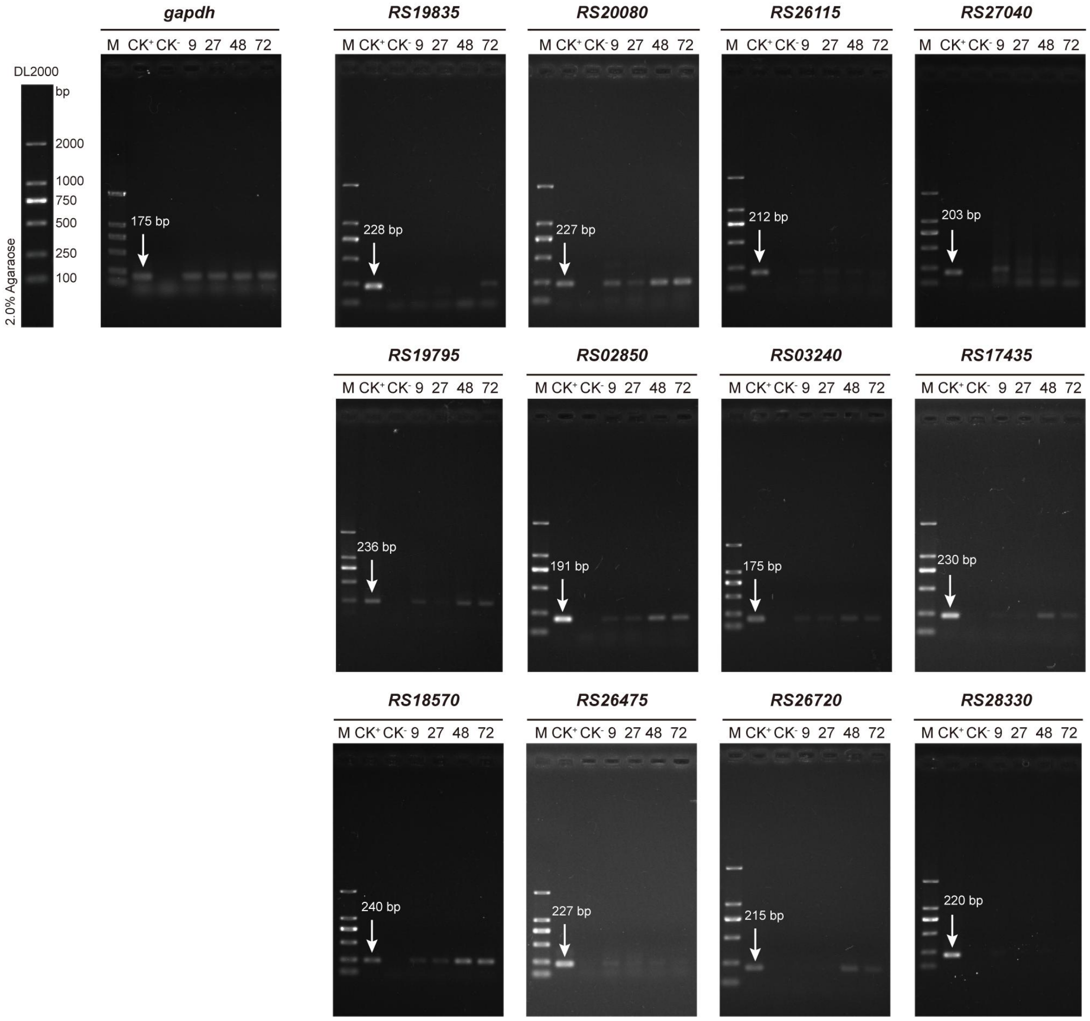

Figure S3. Transcript levels of the twelve genes coding putative proteins at four different sampling time points (9 h, 27 h, 48 h and 72 h) in LB medium .
