# Supplementary material for: c-di-GMP Regulates Various Phenotypes and Insecticidal Activity of Gram-Positive Bacillus thuringiensis
Source: Front Microbiol. 2018 Feb 13;9:45. doi: 10.3389/fmicb.2018.00045 (PMC5816809; doi:10.3389/fmicb.2018.00045)
Supplement: Supplementary file 9 [file Image4.pdf]

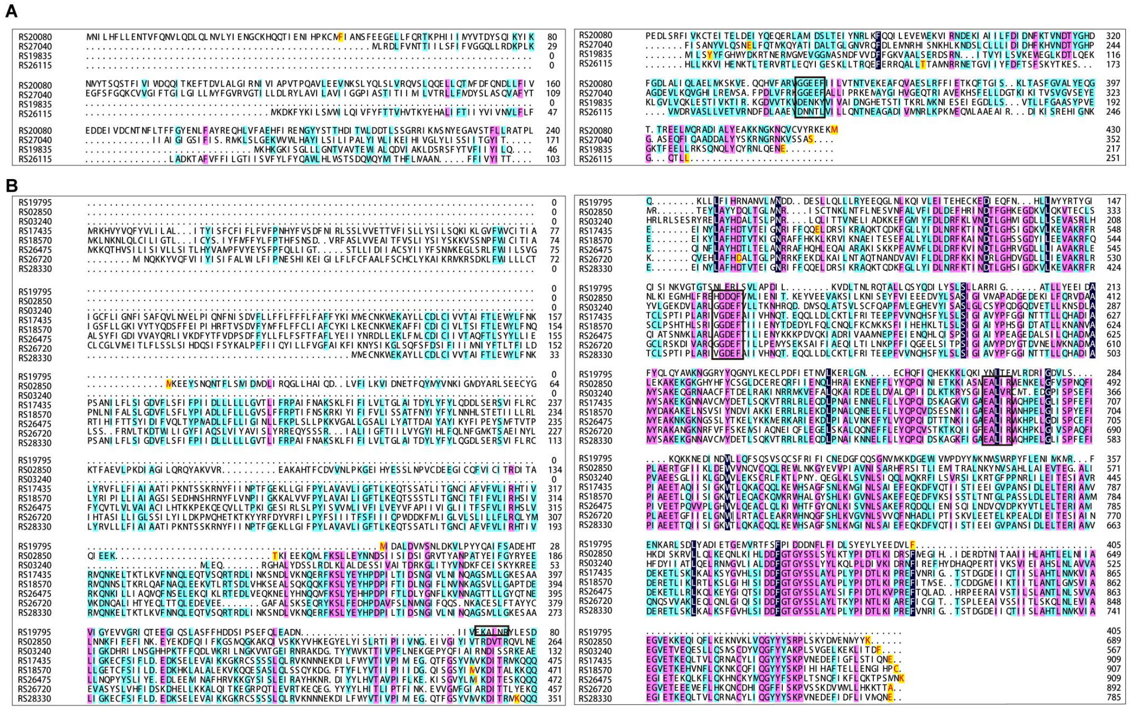

Figure S4. Amino acid sequence alignment of twelve putative proteins and residue boundaries for the domain constructs used for protein expression. (A) Amino acid sequence alignments and residue boundaries used for protein expression for four putative proteins with GGDEF domain. **(B)** Amino acid sequence alignments and residue boundaries used for protein expression for eight putative proteins with an EAL or a dual GGDEF-EAL domain. Black colour shading highlights 100% homology level. Pink colour shading highlights 75% homology level. Blue colour highlights 50% homology level. Red letter and yellow colour shading highlights residue boundaries used for protein expression. Black box highlights probable GGDEF motif and EAL motif.
