# Supplementary material for: c-di-GMP Regulates Various Phenotypes and Insecticidal Activity of Gram-Positive Bacillus thuringiensis
Source: Front Microbiol. 2018 Feb 13;9:45. doi: 10.3389/fmicb.2018.00045 (PMC5816809; doi:10.3389/fmicb.2018.00045)
Supplement: Supplementary file 10 [file Image5.pdf]

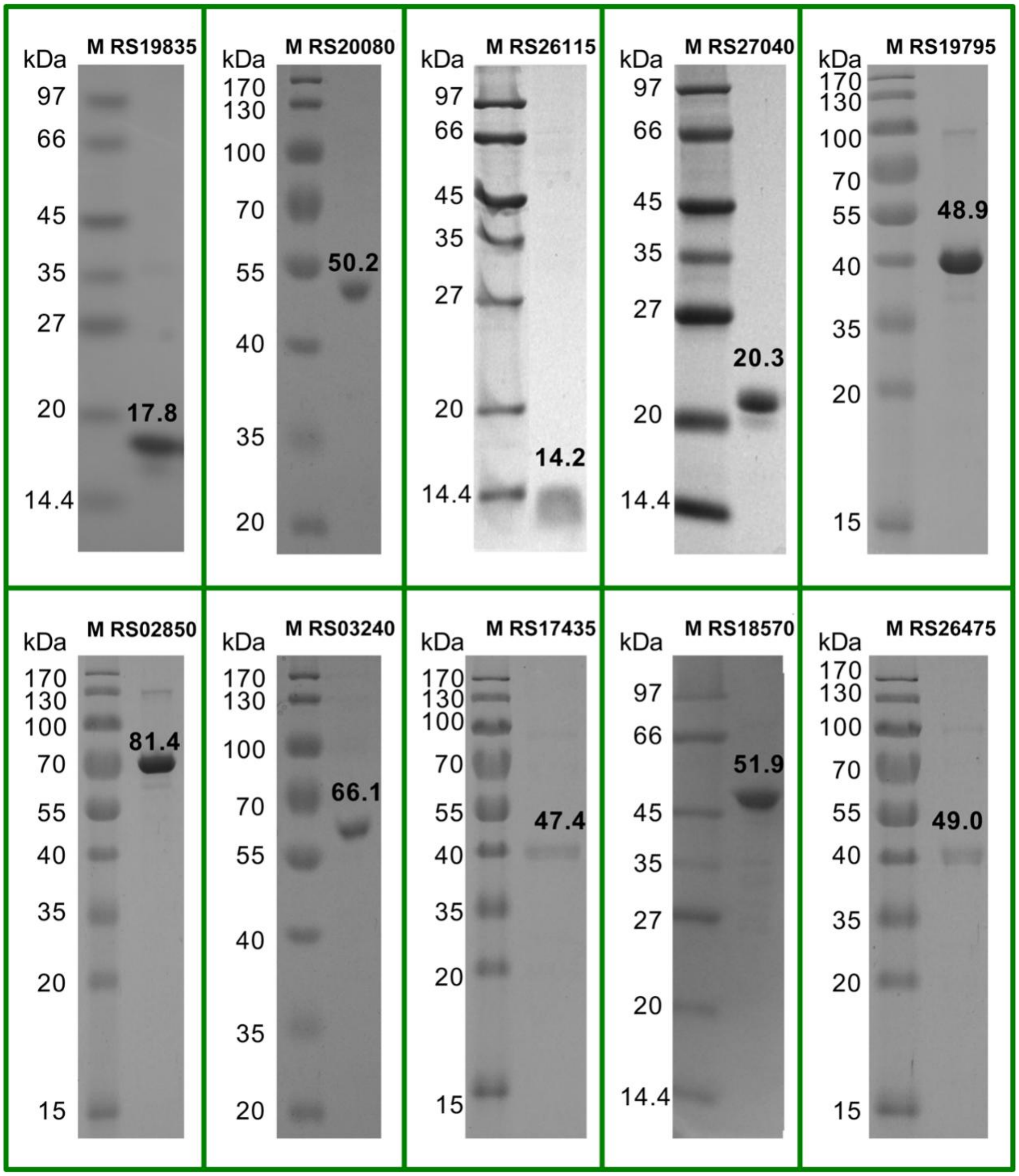

Figure S5. SDS-PAGE analyses of the expression levels of ten His_6-tagged proteins. Lane M: protein molecular weight marker; Lanes 1-10: SDS-PAGE of purified ten proteins containing a GGDEF domain and/or an EAL domain with a C-terminal His_6-tag.
