# Supplementary material for: c-di-GMP Regulates Various Phenotypes and Insecticidal Activity of Gram-Positive Bacillus thuringiensis
Source: Front Microbiol. 2018 Feb 13;9:45. doi: 10.3389/fmicb.2018.00045 (PMC5816809; doi:10.3389/fmicb.2018.00045)
Supplement: Supplementary file 11 [file Image6.pdf]

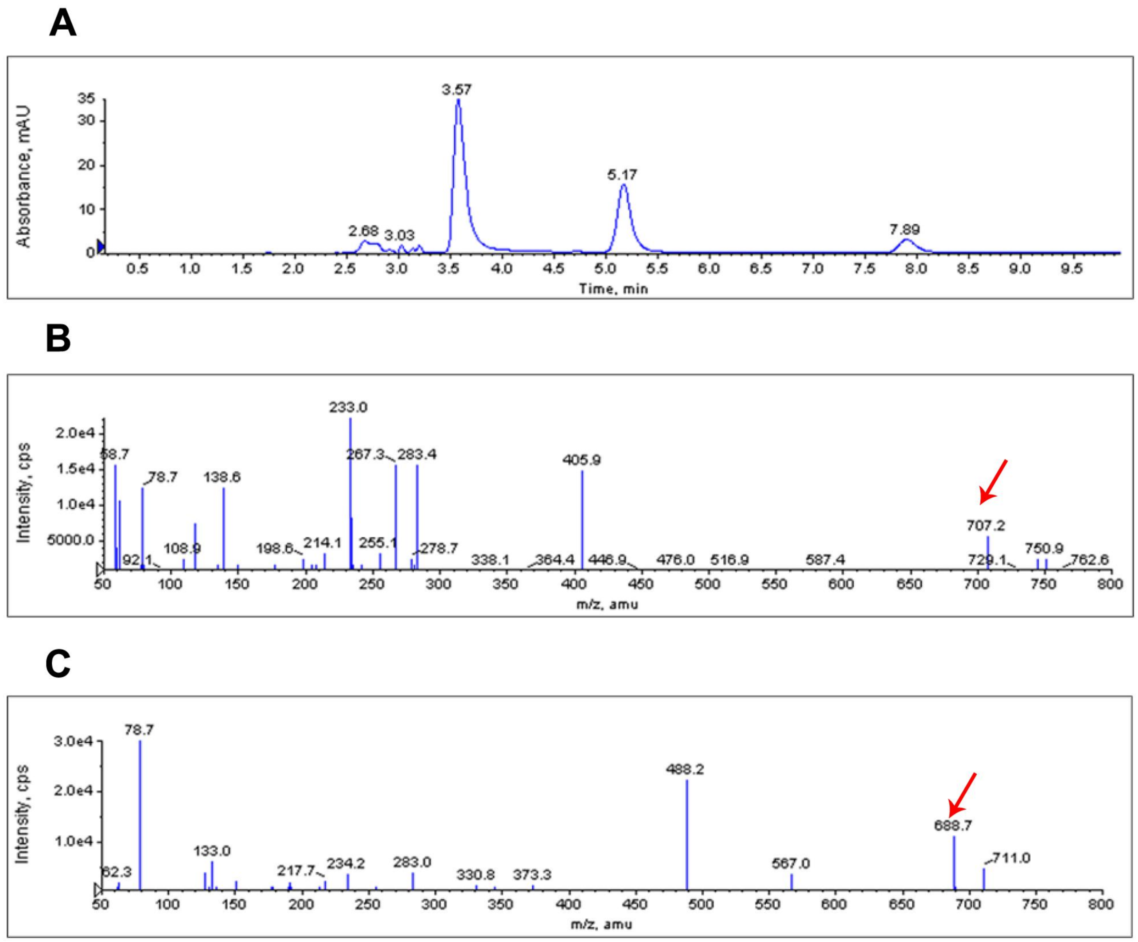

Figure S6. Detection of pGpG and c-di-GMP from reaction mixture of RS03240 by LC-MS analysis. (A) HPLC chromatogram of the reaction production of RS03240. **(B)** The mass spectra of the reaction production of RS03240 at 3.57 min. The red arrow indicated the molecular ion peak corresponding to pGpG at 3.57 min with a *m/z* of 707.2 $[M-H]^-$. **(C)** The mass spectra of the reaction production of RS03240 at 5.17 min. The red arrow indicated the molecular ion peak corresponding to c-di-GMP at 5.17 min with a *m/z* of 688.7 $[M-H]^-$.
