# Supplementary material for: c-di-GMP Regulates Various Phenotypes and Insecticidal Activity of Gram-Positive Bacillus thuringiensis
Source: Front Microbiol. 2018 Feb 13;9:45. doi: 10.3389/fmicb.2018.00045 (PMC5816809; doi:10.3389/fmicb.2018.00045)
Supplement: Supplementary file 12 [file Image7.pdf]

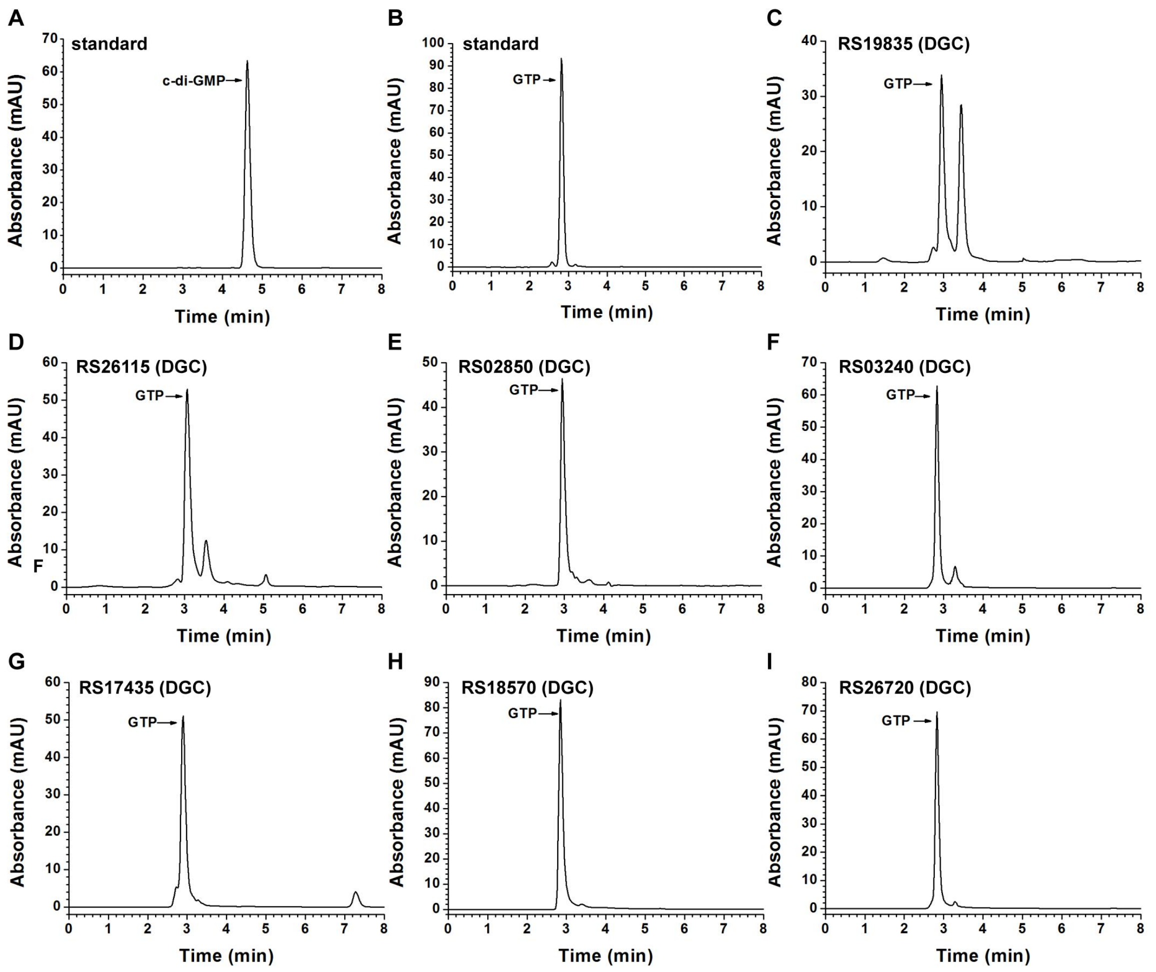

Figure S7. Determination of *in vitro* DGC activities of putative proteins with GGDEF domain. (A to I) The chromatograms of HPLC analyses shown for c-di-GMP standard, GTP standard, single GGDEF domain proteins (RS19835 and RS26115), as well as dual GGDEF-EAL domain proteins (RS02850, RS03240, RS17435, RS18570 and RS26720), respectively.
