# Supplementary material for: c-di-GMP Regulates Various Phenotypes and Insecticidal Activity of Gram-Positive Bacillus thuringiensis
Source: Front Microbiol. 2018 Feb 13;9:45. doi: 10.3389/fmicb.2018.00045 (PMC5816809; doi:10.3389/fmicb.2018.00045)
Supplement: Supplementary file 13 [file Image8.pdf]

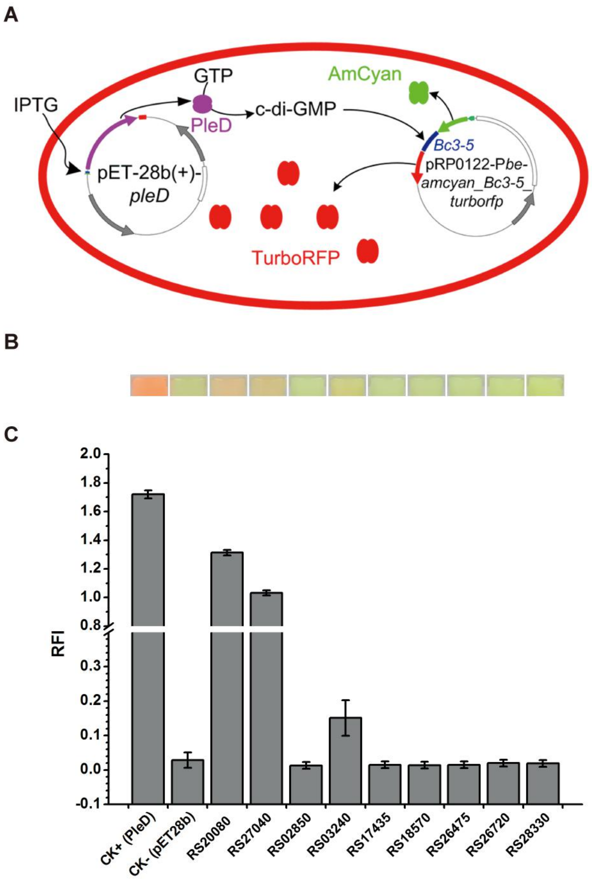

Figure S8. Determination of DGC activity *in vivo* for proteins with a GGDEF domain by using a c-di-GMP biosensor. (A) Schematic diagrams of *amcyan-triple-turborfp* sandwich like dual-fluorescence reporter (Zhou *et al*., 2016). This is a novel system for detecting c-di-GMP concentration *in vivo* by a triple tandem riboswitch in *B. thuringiensis* subsp. *chinensis* CT-43. The PleD (a positive control DGC, orchid ellipsoid dimer) was induced by IPTG to synthesize c-di-GMP. Accumulating c-di-GMP then triggers the c-di-GMP riboswitch to turn on TurboRFP (red ellipsoid dimer) expression, giving rise to TurboRFP fluorescent intensity change *versus* AmCyan (bright green ellipsoid tetramer), which is irresponsive to c-di-GMP. This reporter can be used to determine the *in vivo* DGCs activity of any putative protein. If the putative protein processes the DGC activity, the dual-fluorescence reporter will emit red light. If the putative protein contains no DGC activity, the reporter will emit green light instead. **(B)** Images detection by the above described biosensor for all proteins with a GGDEF domain in BMB171. **(C)** Quantitation of the relative fluorescence intensity of proteins with a GGDEF domain by using a fluorospectrophotometer.
