# Supplementary material for: c-di-GMP Regulates Various Phenotypes and Insecticidal Activity of Gram-Positive Bacillus thuringiensis
Source: Front Microbiol. 2018 Feb 13;9:45. doi: 10.3389/fmicb.2018.00045 (PMC5816809; doi:10.3389/fmicb.2018.00045)
Supplement: Supplementary file 14 [file Image9.pdf]

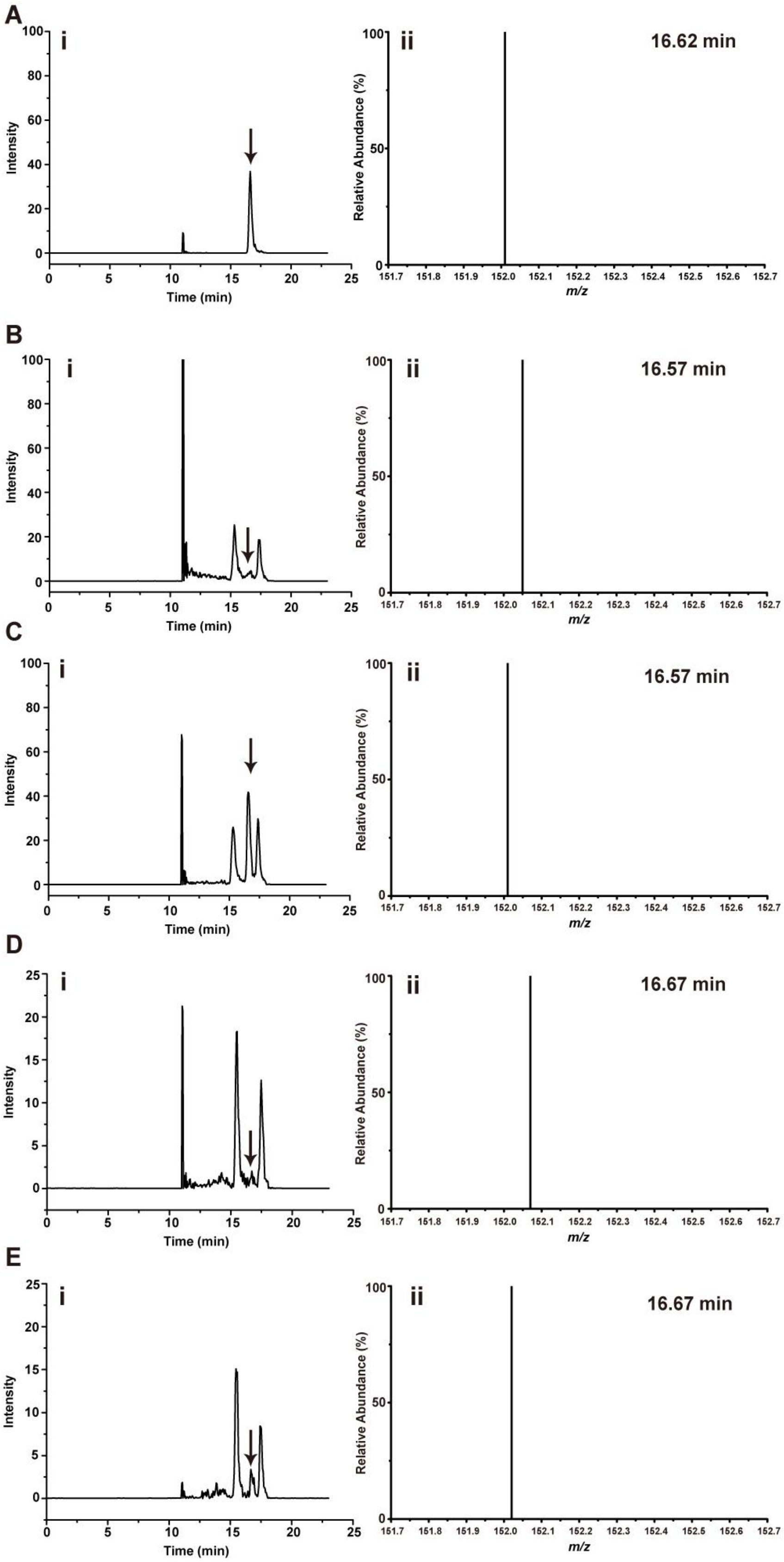
A
i
Intensity
Time (min)
ii
16.62 min
Relative Abundance (%)
m/z
B
i
ii
16.57 min
C
i
ii
16.57 min
D
i
ii
16.67 min
E
i
ii
16.67 min

Figure S9. Detection of intracellular c-di-GMP concentration of BMB171 and Δ3*pde* by LC-MS/MS at different growth phases. (A to E) Quantification of intracellular c-di-GMP concentration by LC-MS/MS for the c-di-GMP standard, BMB171 at 9 h, Δ3*pde* at 9 h, BMB171 at 48 h, and Δ3*pde* at 48 h, respectively. (i) Signal from the select reaction monitor (SRM) *via* LC-MS/MS. (ii) The tandem mass spectra results of the ion fragment of c-di-GMP at *m/z*=691.135/152.000 detected by ESI-MS/MS. The peak of c-di-GMP is marked by the black arrow. And then the peak area of c-di-GMP was calculated using Thermo Xcalibur Qual Browser software and used for counting the concentration of c-di-GMP.
