# Supplementary material for: c-di-GMP Regulates Various Phenotypes and Insecticidal Activity of Gram-Positive Bacillus thuringiensis
Source: Front Microbiol. 2018 Feb 13;9:45. doi: 10.3389/fmicb.2018.00045 (PMC5816809; doi:10.3389/fmicb.2018.00045)
Supplement: Supplementary file 15 [file Image10.pdf]

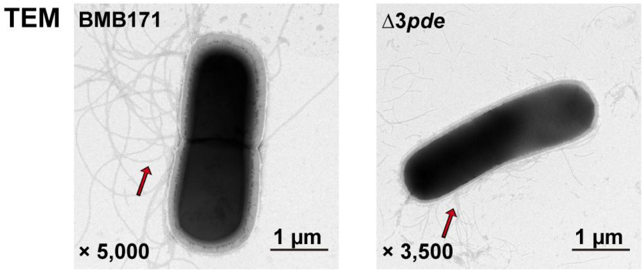

Figure S10. Transmission electron microscope imaging of BMB171 and Δ3*pde* flagella. The bacterial flagella are indicated by red arrows. The strains were streaked on slant semi-solid LB medium containing 0.5% agar and grown at 28°C for 10 h. The cell were resuspended in 1 mL of ddH_2O, and incubated at 37°C for 10 min. A drop of the suspension was pipetted onto a holy carbon-coated copper grid and dried at room temperature. The enlarged TEM micrographs were taken by a FEI Tecnai G20 transmission electron microscope (Hitachi, Tokyo, Japan).
