# Supplementary material for: c-di-GMP Regulates Various Phenotypes and Insecticidal Activity of Gram-Positive Bacillus thuringiensis
Source: Front Microbiol. 2018 Feb 13;9:45. doi: 10.3389/fmicb.2018.00045 (PMC5816809; doi:10.3389/fmicb.2018.00045)
Supplement: Supplementary file 16 [file Image11.pdf]

Figure S6.pdf

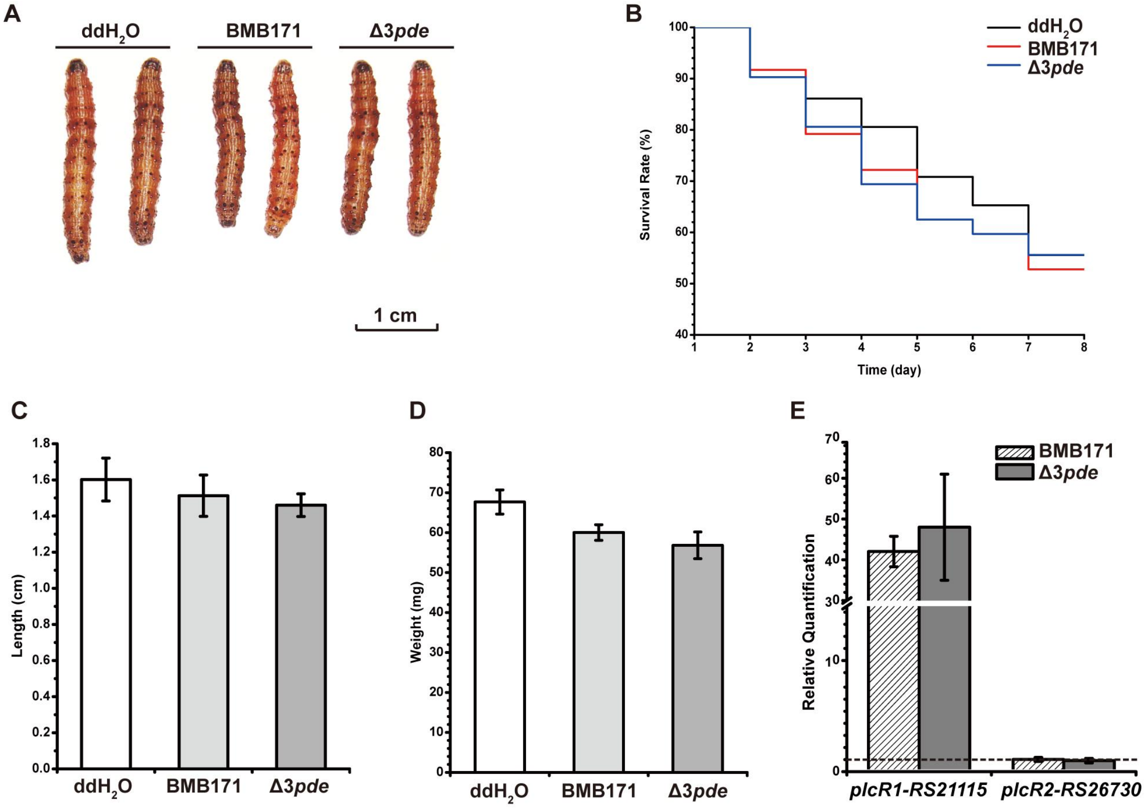

Figure S11. Virulence of BMB171 and Δ3*pde* grown at the exponential phase (9 h) are not affected by increasing c-di-GMP level. (A) The image of cotton bollworm larvae fed with different strains at the end of a rearing cycle. Scale bars represent one centimeter. **(B)** Comparison in survival rates of cotton bollworm larvae fed with BMB171 and Δ3*pde* within rearing cycle (7 days). The survival rate of cotton bollworm larvae fed with Δ3*pde* was almost equal to that fed with BMB171 every day. **(C)** Quantitative measurements of body length of cotton bollworm larvae fed with different *B. thuringenesis* strains. The length of cotton bollworm larvae fed with the Δ3*pde* did not show much difference to those fed with the BMB171. **(D)** Quantitative measurements of body weight of cotton bollworm larvae fed with these different strains. The weight of cotton bollworm larvae fed with the Δ3*pde* did not show a clear change to that fed with the BMB171 too. **(E)** Comparison of relative transcript levels of *plcR1 and plcR2* genes between BMB171 and Δ3*pde* using RT-qPCR method at the mid exponential phase. Error bars represent the standard deviation of three replicates. Dash line represents similar relative quantification. *plcR1* (*RS21115*) and *plcR2* (*RS26730*) did not show conspicuous change in Δ3*pde* compared with that in BMB171.
