# Supplementary material for: c-di-GMP Regulates Various Phenotypes and Insecticidal Activity of Gram-Positive Bacillus thuringiensis
Source: Front Microbiol. 2018 Feb 13;9:45. doi: 10.3389/fmicb.2018.00045 (PMC5816809; doi:10.3389/fmicb.2018.00045)
Supplement: Supplementary file 17 [file Image12.pdf]

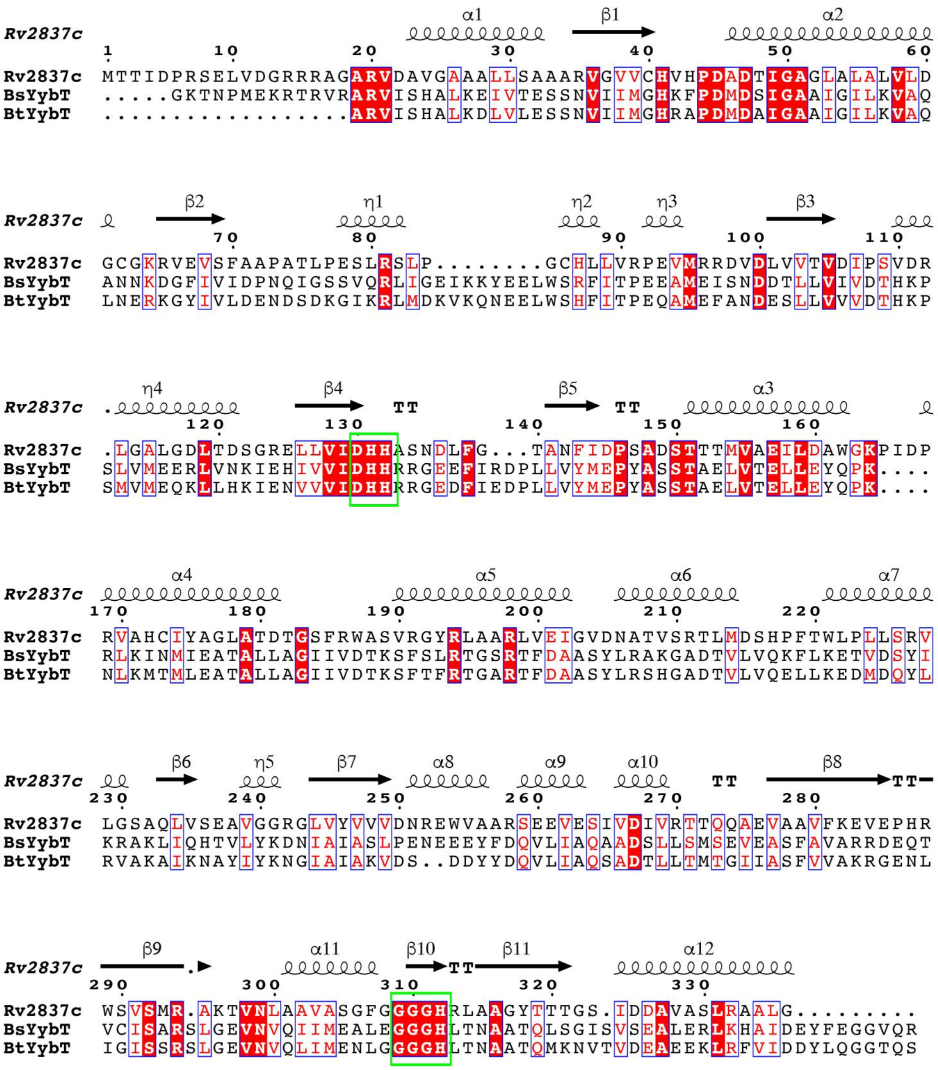

Figure S12. Multiple sequences alignment of YybT. The alignment of *M. tuberculosis* Rv2837c (PDB codes 5CET and 5JJU), *B. subtilis* strain 168 YybT (BsYybT) and *B. thuringiensis* BMB171 YybT (BtYybT) is performed using ESPript 3.0. The secondary structures of Rv2837c are shown at the top of the panel. The amino acids critical for GdpP-like phosphodiesterase activity are highlighted by green boxes.
